# A typology of dietary and anthropometric measures of nutritional need among children across districts and parliamentary constituencies in India, 2016

**DOI:** 10.7189/jogh.10.020424

**Published:** 2020-12

**Authors:** Jacob P Beckerman-Hsu, Pritha Chatterjee, Rockli Kim, Smriti Sharma, S V Subramanian

**Affiliations:** 1 Boston College School of Social Work, Chestnut Hill, Massachusetts, USA; 2 Department of Social and Behavioral Sciences, Harvard T.H. Chan School of Public Health, Boston Massachusetts, USA; 3 Interdisciplinary Program in Precision Public Health, Department of Public Health Sciences, Graduate School of Korea University, Seoul, Republic of Korea; Department of Public Health Sciences, Graduate School, Korea University, Seoul, South Korea; Harvard Center for Population and Development Studies, Cambridge, Massachusetts, USA; 4 Tata Trusts, Delhi, India; 5 Harvard Center for Population and Development Studies, Cambridge, Massachusetts, USA; and Department of Social and Behavioral Sciences, Harvard T. H. Chan School of Public Health, Boston, Massachusetts, USA

## Abstract

**Background:**

Anthropometry is the most commonly used approach for assessing nutritional need among children. Anthropometry alone, however, cannot differentiate between the two immediate causes of undernutrition: inadequate diet vs disease. We present a typology of nutritional need by simultaneously considering dietary and anthropometric measures, dietary and anthropometric failures (DAF), and assess its distribution among children in India.

**Methods:**

We used the 2015-16 National Family Health Survey, a nationally representative sample of children aged 6-23 months (n = 67 247), from India. Dietary failure was operationalized using World Health Organization (WHO) standards for minimum dietary diversity. Anthropometric failure was operationalized using WHO child growth reference standard z-score of <-2 for height-for-age (stunting), weight-for-age (underweight) and weight-for-height (wasting). We also created a combined anthropometric measure for children who had any one of these three anthropometric failures. We cross-tabulated dietary and anthropometric failures to produce four combinations: Dietary Failure Only (DFO), Anthropometric Failure Only (AFO), Both Failures (BF), and Neither Failure (NF). We estimated the prevalence and distribution of the four types, nationally, and across 640 administrative districts and 543 Parliamentary Constituencies (PCs) in India.

**Results:**

Nationally, 80.3% of children had dietary failure and 53.7% had at least one anthropometric failure. The prevalence for the four DAF types was: 44.0% (BF), 36.3% (DFO), 9.8% (AFO), and 9.9% (NF). Dietary and anthropometric measures were discordant for 46.1% of children; these children had nutritional needs identified by only one of the two measures. Nationally, this translates to 12 181 627 children with DFO and 3 281 913 children with AFO; the nutritional needs of these children would not be captured if using only dietary or anthropometric assessment. Substantial variation was observed across districts and PCs for all DAF types. The interquartile ranges for districts were largest for BF (29.8%-53.0%) and lowest for AFO (5.5%-13.4%).

**Conclusions:**

The current emphasis on anthropometry for measuring nutritional need should be complemented with diet- and food-based measures. By differentiating inadequate food intake from other causes of undernutrition, the DAF typology brings precision in identifying nutritional needs among children. These insights may improve the development and targeting of nutrition interventions.

The two immediate causes of undernutrition among children are inadequate dietary intake and disease [1]. Undernutrition can thus be conceptualized as a state of low net nutrition [2], a balance between what children eat and any nutritional losses they experience due to disease. In both research and the discourse that shapes policy agenda, anthropometry, or more specifically, anthropometric failure, is the most commonly used measure of undernutrition [3,4]. Anthropometry, especially height at the individual-level, is a complex indicator that captures genetic, environmental, and behavioural factors, as well as exposure to disease [2]. Even though population-level summaries of World Health Organization (WHO) based definitions of anthropometric failures may be able to track some nutritional deficiencies, they alone cannot be considered a complete measure of low net nutrition because not all nutritional deficiencies would be expected to result in anthropometric failure [5]. For example, globally, among children under-five, Vitamin A deficiency is 30% higher than the prevalence of stunting [6], suggesting many children with a specific nutritional need are not identified as such by anthropometric failure.

Particularly in research, direct assessments of food and dietary intake have been largely overlooked as a measure of nutritional need among children. When utilized, they have been restricted as “explanatory” or “input” variables that contribute to an anthropometric-based measure of undernutrition, such as stunting, underweight, or wasting [7-13]. The use of dietary measures as an “outcome,” or a variable that it is intrinsically important in its own right as an indicator of child nutritional need, is rare in scientific research and the current policy discourse on child undernutrition [14,15].

Dietary measures have two strengths over anthropometry for identifying children in a state of low net nutrition. First, dietary assessment may detect nutritional need overlooked by anthropometry. For instance, the World Health Organization (WHO) minimum dietary diversity indicator was created as a way to use dietary data to capture the micronutrient density of the diets of children 6- 23 months old [16,17]. Children not reaching minimum dietary diversity may therefore be considered as having unmet nutritional need, even if they have no anthropometric failures. This scenario appears to be rather common. For example, among 6-23 month-old children in India, while 36%, 33%, and 24% have stunting, underweight, and wasting, respectively, 78% of the children do not achieve minimum dietary diversity [18]. Other dietary indicators that may capture nutritional need, even among children with no anthropometric failures, include minimum acceptable diet and other WHO Infant and Young Child Feeding (IYCF) indicators [19].

The second advantage to dietary measures is that they can identify nutritional need in a way that is immediately actionable. Dietary data can be used to identify the most pressing food-based needs and evaluate the extent to which those food-based needs have been met by a given policy or program. Such food-based priority setting is especially appropriate given that food supplementation programs are a primary means of addressing undernutrition [20] (eg, Integrated Child Development Services program in India [21]).

At the same time, dietary measures alone cannot be considered a complete indicator of undernutrition because even with an optimal diet, nutritional losses can result in a state of low net nutrition. Given the unique strengths of the well-established anthropometric and dietary diversity-based measures, their simultaneous use has potential to yield insights into nutritional need not made clear by either indicator alone.

We propose a four-category typology of nutritional need based on simultaneously considering “*Dietary and Anthropometric Failures*” (DAF) (**Table 1**). To mirror the concept of anthropometric failure (ie, children with a z score of <-2 on the WHO child growth reference standard on any anthropometric measure of height and weight), we use the term “dietary failure” to refer to children not reaching minimum dietary diversity. Children with “*Both Failures*” (BF) have high unmet needs in terms of intake, and may also have high nutritional losses. Overall, they may have the greatest need, likely requiring both nutrition-specific and nutrition-sensitive approaches [22]. Children with need as measured by “*Anthropometric Failure Only*” (AFO) (ie, with no dietary failure) may have greater needs related to nutritional losses, especially compared to their peers with similar diets and no anthropometric failures. It is also possible that these children have low intake of key nutrients or calories despite their relatively high dietary diversity, resulting in anthropometric failure. Children with need as measured by “*Dietary Failure Only*” (DFO) (ie, who have no anthropometric failures) may have important nutritional deficiencies that have little impact on body size or have yet to impact body size. Finally, children with “*Neither Failure*” (NF) would be expected to have the least unmet nutritional need.

**Table 1:** Interpretation of the four types of dietary and anthropometric failures

	Anthropometric failure	No anthropometric failure
	**Both failures (BF)**	**Dietary failure only (DFO)**
**Dietary failure**	• Low intake of key nutrients	• May have low intake of nutrients with little impact on body size
	• May have high nutritional losses	• Overlooked by anthropometric indicators of undernutrition
	• Highest amount of unmet nutritional need	Nutrition-specific intervention may be most needed (eg, providing supplemental food)
	**Anthropometric failure only (AFO)**	**Neither failure (NF)**
**No dietary failure**	• May have high nutritional losses and/or low intake of key nutrients or calories, resulting in anthropometric failure despite meeting dietary standards • Nutrition-sensitive intervention may be most needed (eg, sanitation)	• Lowest amount of unmet nutritional need

Using this conceptual typology, we empirically examined the prevalence and distribution of all four DAF categories among children in India. We also examined the discordance between dietary and anthropometric failures to understand the degree to which DAF types can highlight needs that cannot be characterized as fully using anthropometry or dietary measures alone. Further, to enable policy-relevant analysis of nutrition and anthropometric failures, we estimate DAF types across two important geographic units in the country. The first is India’s key administrative unit of “districts,” the level at which nutrition policies are planned, executed, and monitored as seen most recently with the country’s ‘aspirational districts program’ [23]. The second is the electoral and political unit of Parliamentary Constituencies (PCs), at which citizens directly elect their representatives and can hence hold them accountable for important policy outcomes in India’s parliamentary system of democracy [24-26].

## METHODS

### Data

The National Family Health Survey 2015-16 (NFHS-4) is a survey on population, health, and nutrition in India that provides representative statistics at the national, state, and district levels. Further detail on the NFHS-4 can be found elsewhere (www.dhsprogram.com). Data can be accessed by request through this source. We also use population estimates from the Spatial Repository Program of the Demographic Health Surveys (DHS) Program [27], to estimate the national burden of each of these indicators in India in 2015.

### Study population and sample size

This analysis includes only children 6-23 months of age to match the criteria for the WHO IYCF indicators [28]. There were 73 093 women with at least one child 6-23 months of age, of whom, 73 068 lived with their youngest child. IYCF indicators are only calculated for the mother’s youngest child living with her [29]. Of this sample, 67 276 children had height and weight measurements. An additional 29 children were dropped because they had no dietary data, yielding a final sample of 67 247.

### Indicators of nutritional need

#### Anthropometric failure

Children were weighed with an electronic SECA 874 flat scale designed for mobile use. For very young children, the mother was weighed alone and then weighed again holding her child. The child’s weight was calculated with an automatic two-in-one adjustment button. Child recumbent length was measured with a Seca 417 infantometer.

Children were categorized as stunted, wasted, or underweight when they had a Z-score below −2 for height-for-age, weight-for-height, or weight-for-age, respectively, relative to WHO standards. These indicators reflect deficiencies in diet and/or disease on chronic (stunting), acute (wasting), and chronic and/or acute (underweight) time scales [30,31]. We also created a composite measure distinguishing children with no anthropometric failures from those with at least one [31].

#### Dietary failure

Mothers participating in the NFHS-4 were asked if their children consumed a variety of foods in the previous day or night, as is standard in the DHS conducted in over 90 countries globally. Responses were used to determine if each child had at least one food from eight food groups: 1) breastmilk (one survey item: currently breastfeeding), 2) grains, roots, and tubers (three survey items: fortified baby food; bread, noodles, or other food made from grains; potatoes, cassava, or other tubers), 3) beans, peas, lentils, nuts (one survey item), 4) dairy (four survey items: tinned, powdered, or fresh milk; baby formula; cheese, yogurt, or other milk products; yogurt), 5) flesh foods (four survey items: chicken, duck, or other birds; liver, heart, or other organs; fish or shellfish; any other meat), 6) eggs (one survey item), 7) vitamin A-rich fruits or vegetables (three survey items: pumpkin, carrots, squash (yellow or orange inside); dark green leafy vegetables; mangoes, papayas, or other vitamin A fruits); and 8) other fruits and vegetables (one survey item: any other fruits). For all foods, answers of “don’t know” were assumed to be “no,” as recommended [29]. Between 27 (bread, noodles, and other food made from grains) and 74 (baby formula) mothers responded with “don’t know.” Given the sample of over 67 000 mothers, this assumption is unlikely to have an important impact on results.

WHO 2010 guidelines for minimum dietary diversity among children aged 6- 23 months did not count breastmilk as a food group [19]. In 2017, when setting targets for 2025, the WHO added breastmilk as an eighth food category, defining minimum dietary diversity as consuming food from five or more of the eight food groups [28]. Because the older and the newer criteria yielded similar results, we present results with the newer criteria.

### Analyses

We calculated the prevalence of all anthropometric and dietary failures, as well as the four DAF categories, at the national, district, and PC levels. We conducted all analyses on RStudio version 1.2.5033. We quantified the discordance between dietary and anthropometric failures as the percent of all children classified differently by both indicators (ie, with anthropometric failure only or dietary failure only).

For deriving PC-level estimates of DAF from district-level data in NFHS, we applied a recently developed and validated method on building a crosswalk between districts and PCs in India [24-26].

Finally, we estimated the burden of the four DAF types using estimates of the national population of children aged 0-4 years old from DHS, 2015 [27]. Since the NFHS is a nationally representative data set, we extrapolated the proportion of children who were aged 6-23 months from the total population of 0-4-year old children in the NFHS (28%), to the national population of children between 0-4 years old as per DHS estimates (N = 117 449 788), to obtain the national population of children of age 6-23 months in India in 2015 (N = 33 535 755). We then estimated the national burden of DAF types in this group in 2015, as per the national percentage of DAF types we identified in the NFHS sample.

### Ethical statement

The Harvard T.H. Chan School of Public Health Institutional Review Board reviewed this study and considered it exempt from full review because it utilizes an anonymous public use data set with no identifiable information on study participants.

## RESULTS

### Typology of Dietary and Anthropometric Failures

**Figure 1:**
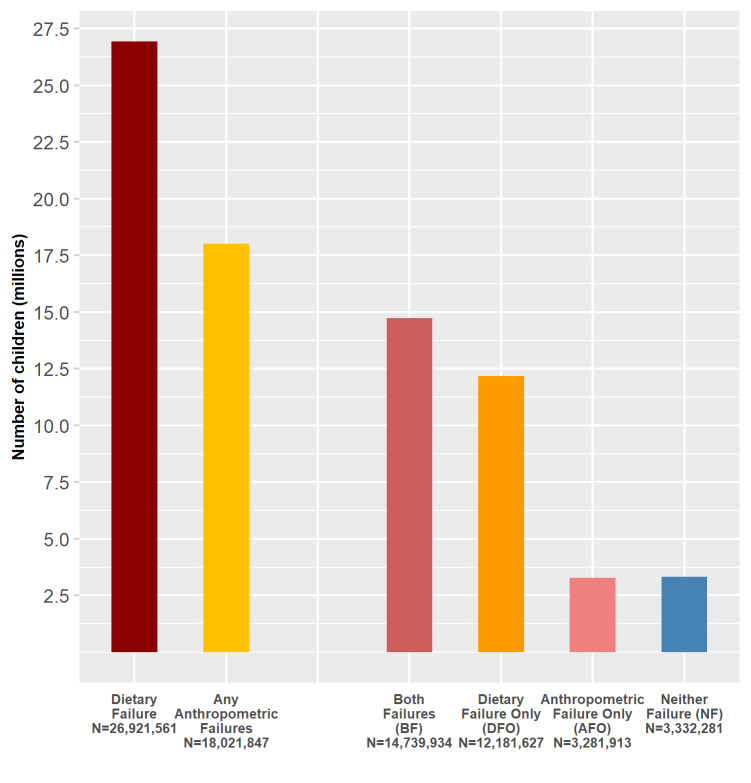
National estimates of population counts in four types of dietary and anthropometric failures among children 6-23 months old in India.

Nationally, 53.7% of children had at least one anthropometric failure and 80.3% had dietary failure. The most common DAF category was BF (prevalence = 44.0%, 95% confidence interval (CI) = 43.6% to 44.3%), followed by DFO (prevalence = 36.3%, 95% CI = 35.9% to 36.7%), NF (prevalence = 9.9%, 95% CI = 9.7% to 10.2%), and AFO (prevalence = 9.8%, 95% CI = 9.5% to 10.0%) (**Table 2**). These prevalences were used to estimate the number of children in each category nationally: BF (N = 14 739 934, 95% CI = 14 607 504 to 14 872 363), DFO (N = 12 181 627, 95% CI = 12 053 063 to 12 310 191), NF (N = 3 332 281, 95% CI = 3 252 151 to 3 412 411), and AFO (N = 3 281 913, 95% CI = 3 200 796 to 3 363 029) (**Figure 1**). In 46.1% of the children, dietary and anthropometric measures of undernutrition were discordant such that children had a dietary but not an anthropometric failure, or vice versa (**Table 2**).

**Table 2:** Frequency and percent of children aged 6-23 months in each type of dietary and anthropometric failures in India*

		Any anthropometric failure			
		**Presence**	**Absence**		**Total**
**Dietary failure**	**Presence**	**Both failures**	**Dietary failure only**		
		29 557 (44.0%)	24 427 (36.3%)		53 984 (80.3%)
	**Absence**	**Anthropometric failure only**	**Neither failure**		
		6581 (9.8%)	6682 (9.9%)		13 263 (19.7%)
Total		36 138 (53.7%)	31 109 (46.3%)		67 247
**Discordance**		46.1%			

Discordance between the three measures of anthropometric failure and dietary failure ranged from 58.1% to 65.0% (**Table 3**). Fewer children had AFO compared to children with DFO; 3.8% were wasted and had no dietary failure, 6.9% were stunted and had no dietary failure, and 5.2% were underweight and had no dietary failure, while 61.3% were not wasted and had dietary failure, 51.2% were not stunted and had dietary failure, and 53.7% were not underweight and had dietary failure (**Table 3**).

**Table 3:** Percent and frequency of children with dietary and anthropometric failures amongst all children aged 6-23 months in India*

		Stunting		Underweight		Wasting		Total
**Presence**	**Absence**	**Presence**	**Absence**	**Presence**	**Absence**
**Dietary failure**	**Presence**	19 528 (29.0%)	34 456 (51.2%)	17 882 (26.6%)	36 102 (53.7%)	12 789 (19.0%)	41 195 (61.3%)	53 984 (80.3%)
	**Absence**	4642 (6.9%)	8621 (12.8%)	3523 (5.2%)	9740 (14.5%)	2532 (3.8%)	10 731 (16.0%)	13 263 (19.7%)
**Total**		24 170 (35.9%)	43 077 (64.1%)	21 405 (31.8%)	45 842 (68.2%)	15 321 (22.8%)	51 926 (77.2%)	67 247 (100.0%)
**Discordance**		58.1%		58.9%		65.0%		

### Geographic distribution of DAF types across districts and PCs

**Figure 2:**
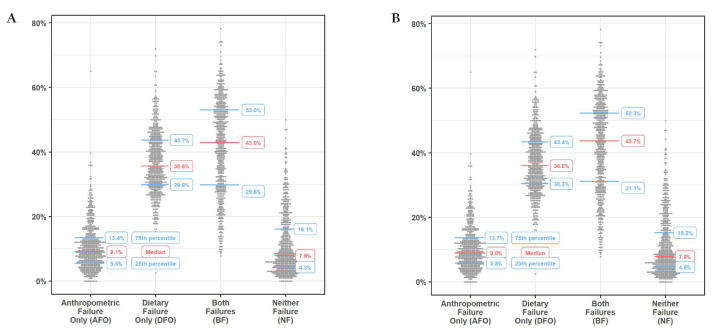
Percentage of types of dietary and anthropometric failures by districts (**A**) and parliamentary constituencies (**B**) in India.

Between 14.4% and 82.2% of children had one or more anthropometric failures and 15.0%-100% had dietary failure at the district level (Table S1 in the **Online Supplementary Document**). Similar patterns were observed for PCs (Table S2 in the **Online Supplementary Document**). Significant heterogeneity was observed between districts and PCs across the DAF types. At the district level, the interquartile ranges (IQR) for DAF types were 29.8%-53.0% for BF, 29.9%-43.7% for DFO, 5.5%-13.4% for AFO, and 4.3%-16.1% for NF (**Figure 2**, Panel A). Similar trends in IQRs were observed at the PC level (**Figure 2**, Panel B).

Districts in the top decile for two key types of DAF, namely, DFO and AFO, are identified as high focus districts (**Table 4**). Children in these categories have nutritional needs that would be overlooked if only anthropometric or dietary data were used. For DFO, several high focus districts were from the northern part of the country in states like Himachal Pradesh, Haryana, Uttar Pradesh, and Rajasthan. For AFO, many high focus districts were situated in southern states like Tamil Nadu and Kerala, and in northeastern states like Meghalaya (**Table 4**).

**Table 4:** High focus districts in the top decile for dietary failure only (DFO) and anthropometric failure only (AFO) in India

DFO		AFO	
**District**	**State**	**District**	**State**
Nicobars, South Andaman	Andaman and Nicobar Islands	North & Middle Andaman	Andaman and Nicobar Islands
Chittoor, Guntur, West Godavari	Andhra Pradesh	Anjaw, Lower Dibang Valley, Lower Subansiri	Arunachal Pradesh
East Siang	Arunachal Pradesh	Dibrugarh, Golaghat	Assam
Dima Hasao	Assam	Baramula, Doda Kargil, Punch, Rajouri, Ramban, Srinagar	Jammu and Kashmir
Chandigarh	Chandigarh	Simdega	Jharkhand
North Goa	Goa	Chikmagalur	Karnataka
Porbandar, Vadodara	Gujarat	Kollam, Kozhikode, Malappuram, Thiruvananthapuram	Kerala
Rewari	Haryana	Yavatmal	Maharashtra
Bilaspur, Kinnaur, Kullu. Lahul and Spiti, Mandi, Sirmaur, Una	Himachal Pradesh	East Garo Hills, East Khasi Hills, Ribhoi, South Garo Hills, West Garo Hills, West Khasi Hills	Meghalaya
Anantnag, Badgam, Shupiyan	Jammu and Kashmir	Kiphire	Nagaland
Hassan	Karnataka	Kendujhar, Subarnapur	Odisha
Alappuzha, Palakkad. Pathanamthitta	Kerala	Mahe, Puducherry, Yanam	Puducherry
Mandsaur	Madhya Pradesh	South District, West District	Sikkim
Bhandara, Kolhapur, Mumbai Suburban	Maharashtra	Cuddalore, Dharmapuri, Dindigul, Erode, Kancheepuram, Karur, Krishnagiri, Madurai, Nagapattinam, Namakkal, Perambalur, Pudukkottai, Thanjavur, The Nilgiris, Theni, Thiruvallur, Thiruvarur,Thoothukkudi, Tiruchirappalli, Tirunelveli, Tiruppur, Tiruvannamalai, Vellore, Viluppuram, Virudhunagar	Tamil Nadu
Bishnupur	Manipur	Bankura, Barddhaman, Nadia, Paschim Medinipur, Puruliya	West Bengal
Lunglei	Mizoram		
Mokokchung, Peren, Tuensang	Nagaland		
Cuttack, Dhenkanal, Puri	Odisha		
Amritsar, Bathinda, Fatehgarh Sahib, Gurdaspur, Kapurthala, Ludhiana, Moga, Rupnagar, Sahibzada Ajit Singh Nagar, Sangrur, Tarn Taran	Punjab		
Ganganagar, Hanumangarh, Jhunjhunun, Nagaur, Sikar	Rajasthan		
Karimnagar, Mahbubnagar, Nizamabad	Telangana		
North Tripura, South Tripura, West Tripura	Tripura		
Gautam Buddha Nagar, Ghaziabad, Saharanpur	Uttar Pradesh		
Garhwal	Uttarakhand		

**Figure 3:**
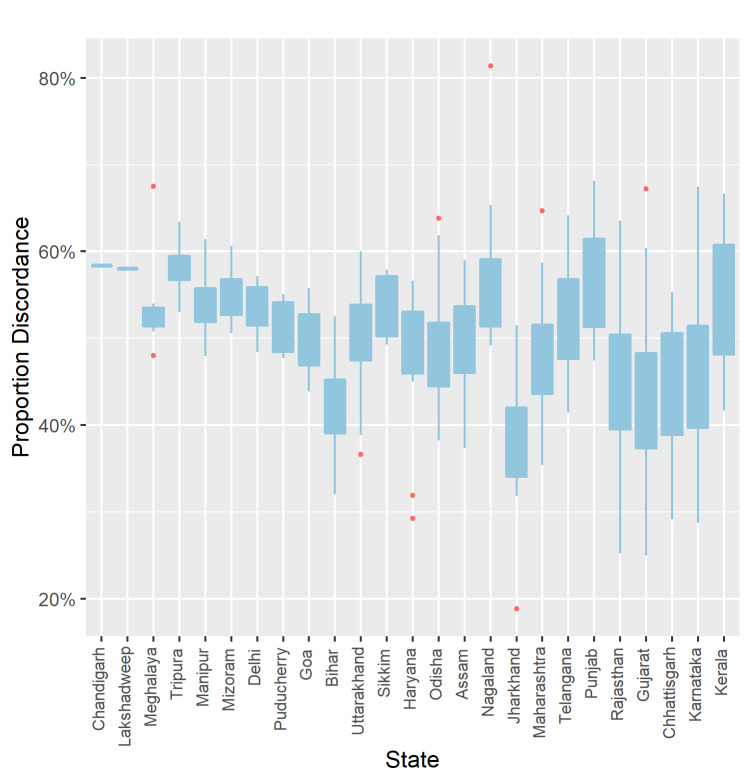
District level discordance between dietary and anthropometric failures by states in India. Box shows interquartile range. Whiskers reach up to 1.5 times interquartile range from the first and third quartiles. Remaining points are plotted individually. States are ordered from lowest to highest interquartile range.

The district-level discordance of anthropometric and dietary failures varied considerably by states, with the IQR of district-level discordance in states with more than one district ranging from 1.29% in Daman and Diu to 12.65% in Kerala **(****Figure 3**). Complete data on percentage of DAF types and their discordance across districts and PCs is included as Table S1 and S2 in the **Online Supplementary Document**. Complete ranking of DAF types across districts and PCs are presented in Table S3 and S4 in the **Online Supplementary Document**.

## DISCUSSION

Our study has three main findings. First, over one third of infants and young children in India have no anthropometric failures, yet do have dietary failure (DFO). This amounts to over 12 million children 6 - 23 months of age across the country with unmet dietary need who are not identified as such by measures focused solely on anthropometry. Second, about 4 in 10 children had both dietary and anthropometric failures, underscoring the depth of nutritional need among a large proportion of the population. Finally, there is substantial geographic heterogeneity in DAF by districts and PCs suggesting the need to consider the area-specific types of nutritional needs for effective and equitable program and policy targeting. The four DAF categories can provide greater insight into the nature of the nutritional need than can dietary or anthropometric indicators alone. Specifically, children with DFO stand to have improved nutritional status with a more diverse diet, even if it has no effect on their physical size, while children with AFO may benefit more from efforts focused on non-diet based interventions.

Although both diet and anthropometry have important functions in the area of undernutrition, they are seldom used in tandem. Dietary measurement has been used to evaluate important nutrition initiatives such as those of Alive and Thrive [32], and anthropometric measures are central to global and national nutrition monitoring efforts [28]. Yet, particularly in the area of monitoring, diet is often considered a process measure to be targeted on the path to addressing anthropometric failure [28], even though, as we showed, both diet and anthropometry can provide insights into distinct aspects of the same underlying issue of undernutrition. Similarly, in studies of undernutrition, dietary diversity is often a predictor of anthropometric failure rather than an outcome studied alongside anthropometric failure [7-13].

We exemplify below how the integration of dietary assessment and anthropometric assessment can quantify and characterize nutritional need at the community level in ways not possible using anthropometry alone.

In the district of Kullu in the state of Himachal Pradesh, nearly three quarters of children (72.0%) have DFO. This district would be considered low-need if anthropometry were the only measure of undernutrition used, but the DAF typology indicates clear need for increased dietary diversity. As such, nutrition-specific interventions [22], like provision of supplemental foods through the Integrated Child Development Services program, may be appropriate. It is also important to note that DAF typology is likely to be more sensitive than anthropometric failure to the impacts of such an intervention in Kullu. Because most of the children in Kullu have no anthropometric failures (74.7%), anthropometric indicators will not capture meaningful changes in nutrition status for the vast majority of children in this district.

Another important example of how a DAF typology can direct intervention efforts is the district of South Garo Hills in the state of Meghalaya. In this district, nearly two thirds of children had AFO (65.0%). The high prevalence of anthropometric failure quickly identifies this district as one with high nutritional needs. While children in South Garo Hills may benefit from nutrition-specific interventions [22] like supplemental foods, nutritional losses may be what separate them from their peers with similarly diverse diets who have no anthropometric failures. If this were the case, supplementary food programs likely would not have substantial impact in South Garo Hills. Instead, it may be more appropriate to focus on minimizing nutritional losses through nutrition-sensitive programs [22] such as sanitation and health care services. It may also be important to do further work to understand calorie and nutrient intake insufficiency in the population, as children not categorized as having dietary failure may have insufficient intake. Similar to Kullu, a DAF typology may help to prioritize intervention efforts for addressing nutritional need in South Garo Hills.

While policies in India are implemented and monitored at the administrative level of districts, the PC analysis highlights estimates of the DAF types at a geographic unit that is entwined with political accountability in the country. In both smaller and larger states, a number of PCs have high rates of DFO, indicating the need for urgent political attention and commitment for nutrition specific measures to galvanize policy action. For example, Silchar constituency in Assam, a relatively small state, had an AFO prevalence of 3.6% and a DFO prevalence of 47.0%, and Muzaffarnagar PC in India’s largest state, Uttar Pradesh, had an AFO prevalence of 4.7% and a DFO prevalence of 39.8%. On the other hand, Tiruvannamalai in Tamil Nadu state has a high AFO of 33.6%, compared to a DFO of 14.0%, indicating the need for more nutrition sensitive policies to meet the nutritional needs of this constituency. As illustrated with these examples, the PC estimates should provide elected representatives a contextual and relevant geographical unit to identify the unique nutritional needs of their electorate, and advocate for the most applicable interventions. At the same time, these estimates provide citizens of India an evidence base to hold their elected representatives accountable for ushering in policies for their specific nutritional needs [26,33].

We outline two main data limitations. First, measurement error may bias estimates of prevalence. A strength of anthropometric data in this study is that it was directly measured with standardized equipment, minimizing many potential sources of bias. Dietary measurement, on the other hand, is prone to recall and other types of bias. However, the dietary measures used in this study have been found appropriate for population-level assessment of dietary intake and are widely used [19]. Moreover, dietary measurement is non-invasive and inexpensive, especially relative to biochemical measurements, which are the other primary measures of nutritional need apart from anthropometry [3]. As such, measuring DAF types as we have done in this paper is a more feasible approach than more expensive methods that could provide higher validity. Second, our estimates may be biased by survey non-response and missing data for specific survey items. Because 92.0% of all mothers who participated in the survey had complete data for their child’s dietary and anthropometric variables in our study, the magnitude of any bias induced by missing data are expected to be small.

Future studies are needed to identify the best dietary and anthropometric measures to further improve the DAF typology. For anthropometry, we used the composite of any anthropometric failure to most fully capture the burden of undernutrition. Future work might consider a different combination of these anthropometric measures and/or other anthropometric measures (eg, mid-upper arm circumference, body mass index). For diet, we used the WHO minimum dietary diversity indicator as an example because it was designed to capture the nutrient density of the diets of children 6-23 months old and has been validated for this purpose [16,17,34-36]. Future studies might consider other measures of diet (eg, minimum acceptable diet) or developing new dietary indicators that maximize sensitivity and specificity for nutritional deficiencies not detected by anthropometry. Implementing DAF typology will require dietary measures to be integrated into routine population health surveys alongside anthropometry. Dietary data can be used to monitor unmet nutritional need with the DAF and also to inform and evaluate nutrition programs and policies more broadly, as is done in some high-income countries (eg, [37,38]).

## CONCLUSION

This study demonstrates how currently used anthropometric measures of undernutrition can be augmented with dietary data to identify nutritional need that would otherwise be overlooked and provide greater insights into the nature of that need. The current emphasis on anthropometry should be complemented with data on diet- and food-based measures. By differentiating inadequate food intake from other causes of undernutrition, the DAF typology provides sharper precision in identifying nutritional needs among children, thus allowing development and targeting of interventions in an effective and equitable manner.

## Additional material

Online Supplementary Document
