# Supplementary material for: A typology of dietary and anthropometric measures of nutritional need among children across districts and parliamentary constituencies in India, 2016
Source: J Glob Health. 2020 Aug 24;10(2):020424. doi: 10.7189/jogh.10.020424 (PMC7569191; doi:10.7189/jogh.10.020424)
Supplement: Online Supplementary Document [file jogh-10-020424-s001.zip › Beckerman AU Supplementary Table 3.pdf]

Supplementary Table 3: District level percentage of types of Dietary and Anthropometric Failures by decile in India.

Paper Title: A Typology of Dietary and Anthropometric Measures of Nutritional Need Among Children Across Districts and Parliamentary Constituencies in India, 2016

Suggested citation: Beckerman-Hsu, JP, Chatterjee, P, Kim, R, Sharma, S, Subramanian SV. A Typology of Dietary and Anthropometric Measures of Nutritional Need Among Children Across Districts and Parliamentary Constituencies in India, 2016. *Journal of Global Health.* 2020. doi: 10.7189/jogh.10.020424

Table S3: District level percentage of types of Dietary and Anthropometric Failures by decile in India.

Deciles	1	2	3	4	5	6	7	8	9	10

District	State	Diet Failure only (DFO)	Anthropometric Failure only (AFO)	Both Failures (BF)	Neither Failure (NF)
Nicobars	Andaman and Nicobar Islands	56.9%	13.9%	13.9%	15.3%
North & Middle Andaman	Andaman and Nicobar Islands	25.0%	25.0%	32.7%	17.3%
South Andaman	Andaman and Nicobar Islands	55.9%	11.9%	16.9%	15.3%
Srikakulam	Andhra Pradesh	48.3%	5.0%	36.7%	10.0%
Vizianagaram	Andhra Pradesh	31.4%	12.9%	42.9%	12.9%
Visakhapatnam	Andhra Pradesh	46.5%	14.0%	27.9%	11.6%
East Godavari	Andhra Pradesh	48.4%	6.5%	33.9%	11.3%
West Godavari	Andhra Pradesh	50.0%	7.5%	40.0%	2.5%
Krishna	Andhra Pradesh	46.8%	8.5%	36.2%	8.5%
Guntur	Andhra Pradesh	51.9%	5.6%	25.9%	16.7%
Prakasam	Andhra Pradesh	37.3%	14.7%	29.3%	18.7%
Sri Potti Sriramulu Nellore	Andhra Pradesh	38.0%	12.0%	44.0%	6.0%
Y.S.R.	Andhra Pradesh	32.8%	9.4%	43.8%	14.1%
Kurnool	Andhra Pradesh	39.4%	9.1%	45.5%	6.1%
Anantapur	Andhra Pradesh	30.7%	18.7%	40.0%	10.7%
Chittoor	Andhra Pradesh	52.9%	7.1%	37.1%	2.9%
Tawang	Arunachal Pradesh	45.7%	17.1%	8.6%	28.6%
West Kameng	Arunachal Pradesh	27.5%	15.7%	9.8%	47.1%
East Kameng	Arunachal Pradesh	25.4%	16.4%	40.3%	17.9%
Papumpare	Arunachal Pradesh	40.6%	14.1%	13.3%	32.0%
Upper Subansiri	Arunachal Pradesh	25.4%	17.9%	26.9%	29.9%
West Siang	Arunachal Pradesh	34.0%	11.3%	30.2%	24.5%
East Siang	Arunachal Pradesh	53.3%	11.1%	22.2%	13.3%
Upper Siang	Arunachal Pradesh	35.0%	5.0%	45.0%	15.0%
Changlang	Arunachal Pradesh	45.3%	9.4%	39.1%	6.3%
Tirap	Arunachal Pradesh	27.0%	8.1%	55.4%	9.5%
Lower Subansiri	Arunachal Pradesh	19.5%	29.3%	29.3%	22.0%
Kurung Kumey	Arunachal Pradesh	32.6%	15.7%	36.0%	15.7%
Dibang Valley	Arunachal Pradesh	42.9%	12.2%	34.7%	10.2%
Lower Dibang Valley	Arunachal Pradesh	40.9%	21.2%	19.7%	18.2%
Lohit	Arunachal Pradesh	37.4%	12.1%	37.4%	13.1%
Anjaw	Arunachal Pradesh	24.1%	24.1%	27.6%	24.1%
Kokrajhar	Assam	41.0%	15.7%	27.7%	15.7%
Dhubri	Assam	30.6%	11.7%	50.5%	7.2%
Goalpara	Assam	26.3%	18.2%	39.4%	16.2%
Barpeta	Assam	38.9%	19.4%	32.4%	9.3%
Morigaon	Assam	45.2%	7.9%	35.7%	11.1%
Nagaon	Assam	37.2%	13.2%	34.7%	14.9%
Sonitpur	Assam	38.8%	12.9%	40.0%	8.2%
Lakhimpur	Assam	41.8%	12.2%	26.5%	19.4%
Dhemaji	Assam	41.5%	10.4%	29.2%	18.9%
Tinsukia	Assam	32.3%	11.8%	35.5%	20.4%

Dibrugarh	Assam	32.5%	21.3%	32.5%	13.8%
Sivasagar	Assam	38.6%	14.3%	25.7%	21.4%
Jorhat	Assam	41.5%	12.2%	26.8%	19.5%
Golaghat	Assam	24.4%	23.2%	29.3%	23.2%
Karbi Anglong	Assam	38.9%	18.9%	25.6%	16.7%
Dima Hasao	Assam	49.0%	10.0%	32.0%	9.0%
Cachar	Assam	47.0%	3.6%	49.4%	0.0%
Karimganj	Assam	37.2%	13.2%	39.5%	10.1%
Hailakandi	Assam	36.7%	12.5%	39.2%	11.7%
Bongaigaon	Assam	25.3%	12.0%	42.7%	20.0%
Chirang	Assam	37.7%	6.6%	48.1%	7.5%
Kamrup	Assam	40.2%	13.4%	37.8%	8.5%
Kamrup Metropolitan	Assam	29.4%	10.3%	22.1%	38.2%
Nalbari	Assam	45.5%	3.9%	41.6%	9.1%
Baksa	Assam	33.3%	19.4%	26.9%	20.4%
Darrang	Assam	33.7%	10.5%	50.5%	5.3%
Udalguri	Assam	40.0%	16.7%	36.7%	6.7%
Pashchim Champaran	Bihar	37.6%	12.0%	41.4%	9.0%
Purba Champaran	Bihar	33.3%	7.4%	50.6%	8.6%
Sheohar	Bihar	32.6%	12.5%	49.5%	5.4%
Sitamarhi	Bihar	27.7%	12.5%	56.5%	3.3%
Madhubani	Bihar	35.5%	16.3%	43.6%	4.7%
Supaul	Bihar	30.0%	11.8%	52.7%	5.5%
Araria	Bihar	28.9%	16.4%	47.8%	7.0%
Kishanganj	Bihar	26.7%	13.3%	53.3%	6.7%
Purnia	Bihar	34.4%	12.1%	47.4%	6.0%
Katihar	Bihar	32.0%	7.7%	55.6%	4.7%
Madhepura	Bihar	31.5%	5.1%	59.9%	3.6%
Saharsa	Bihar	32.0%	7.9%	54.2%	5.9%
Darbhanga	Bihar	34.0%	11.3%	45.9%	8.8%
Muzaffarpur	Bihar	34.8%	5.7%	51.1%	8.5%
Gopalganj	Bihar	40.3%	12.2%	38.8%	8.6%
Siwan	Bihar	40.4%	8.6%	43.7%	7.3%
Saran	Bihar	35.4%	10.4%	44.4%	9.7%
Vaishali	Bihar	35.7%	11.1%	47.7%	5.5%
Samastipur	Bihar	26.9%	6.4%	58.5%	8.2%
Begusarai	Bihar	37.1%	7.5%	49.5%	5.9%
Khagaria	Bihar	33.3%	9.9%	51.4%	5.4%
Bhagalpur	Bihar	29.6%	12.8%	52.2%	5.4%
Banka	Bihar	29.4%	9.1%	57.2%	4.3%
Munger	Bihar	30.5%	11.9%	53.0%	4.6%
Lakhisarai	Bihar	30.3%	8.6%	56.2%	4.9%
Sheikhpura	Bihar	21.3%	18.6%	53.7%	6.4%
Nalanda	Bihar	30.8%	11.9%	53.1%	4.2%
Patna	Bihar	31.6%	4.8%	57.7%	5.8%
Bhojpur	Bihar	27.7%	10.4%	54.3%	7.5%
Buxar	Bihar	31.5%	8.4%	50.0%	10.1%
Kaimur (Bhabua)	Bihar	30.6%	6.5%	59.1%	3.8%
Rohtas	Bihar	32.1%	1.2%	63.7%	3.0%
Aurangabad	Bihar	27.2%	12.2%	56.5%	4.1%

Gaya	Bihar	27.3%	13.9%	57.1%	1.7%
Nawada	Bihar	32.7%	13.7%	44.6%	8.9%
Jamui	Bihar	25.7%	10.4%	60.1%	3.8%
Jehanabad	Bihar	27.6%	17.3%	46.8%	8.3%
Arwal	Bihar	26.0%	6.0%	64.5%	3.5%
Chandigarh	Chandigarh	56.3%	2.1%	39.6%	2.1%
Korea (Koriya)	Chhattisgarh	29.4%	11.9%	43.8%	14.9%
Surguja	Chhattisgarh	36.0%	13.5%	37.8%	12.6%
Jashpur	Chhattisgarh	44.7%	10.7%	35.9%	8.7%
Raigarh	Chhattisgarh	41.1%	11.0%	34.2%	13.7%
Korba	Chhattisgarh	39.4%	7.5%	48.7%	4.4%
Janjgir - Champa	Chhattisgarh	36.0%	16.3%	38.4%	9.3%
Bilaspur	Chhattisgarh	34.3%	12.4%	47.6%	5.7%
Kabirdham	Chhattisgarh	32.0%	10.2%	49.2%	8.6%
Rajnandgaon	Chhattisgarh	28.2%	9.4%	52.9%	9.4%
Durg	Chhattisgarh	46.2%	5.4%	43.5%	4.9%
Raipur	Chhattisgarh	40.1%	7.8%	43.8%	8.3%
Mahasamund	Chhattisgarh	34.6%	16.3%	38.5%	10.6%
Dhamtari	Chhattisgarh	32.2%	8.0%	55.2%	4.6%
Uttar Bastar Kanker	Chhattisgarh	32.3%	6.5%	55.9%	5.4%
Bastar	Chhattisgarh	23.4%	9.0%	61.3%	6.3%
Narayanpur	Chhattisgarh	26.6%	2.5%	69.6%	1.3%
Dakshin Bastar Dantewada	Chhattisgarh	26.5%	8.0%	63.7%	1.8%
Bijapur	Chhattisgarh	30.4%	8.9%	58.9%	1.8%
Dadra & Nagar Haveli	Dadra and Nagar Haveli	32.9%	0.0%	67.1%	0.0%
Diu	Daman and Diu	47.8%	4.3%	39.1%	8.7%
Daman	Daman and Diu	47.6%	7.1%	28.6%	16.7%
North Goa	Goa	51.9%	3.8%	30.8%	13.5%
South Goa	Goa	40.4%	3.5%	33.3%	22.8%
Kachchh	Gujarat	31.6%	7.9%	55.3%	5.3%
Banaskantha	Gujarat	35.0%	7.8%	50.5%	6.8%
Patan	Gujarat	47.1%	5.9%	44.1%	2.9%
Mahesana	Gujarat	32.9%	3.9%	57.9%	5.3%
Sabarkantha	Gujarat	42.7%	6.1%	48.8%	2.4%
Gandhinagar	Gujarat	40.9%	3.0%	54.5%	1.5%
Ahmadabad	Gujarat	35.6%	3.4%	54.2%	6.8%
Surendranagar	Gujarat	29.9%	2.6%	67.5%	0.0%
Rajkot	Gujarat	47.0%	4.5%	40.9%	7.6%
Jamnagar	Gujarat	29.1%	10.1%	51.9%	8.9%
Porbandar	Gujarat	56.6%	3.8%	35.8%	3.8%
Junagadh	Gujarat	42.6%	3.3%	50.8%	3.3%
Amreli	Gujarat	42.4%	8.5%	42.4%	6.8%
Bhavnagar	Gujarat	35.5%	4.8%	53.2%	6.5%
Anand	Gujarat	34.3%	4.3%	55.7%	5.7%
Kheda	Gujarat	34.8%	9.0%	52.8%	3.4%
Panchmahal	Gujarat	28.8%	5.5%	61.6%	4.1%
Dohad	Gujarat	27.4%	8.4%	61.1%	3.2%
Vadodara	Gujarat	55.2%	12.1%	31.0%	1.7%
Narmada	Gujarat	22.1%	2.9%	73.1%	1.9%
Bharuch	Gujarat	32.9%	7.1%	54.3%	5.7%

The Dangs	Gujarat	23.2%	8.4%	67.4%	1.1%
Navsari	Gujarat	30.5%	8.5%	52.5%	8.5%
Valsad	Gujarat	37.5%	17.9%	37.5%	7.1%
Surat	Gujarat	38.7%	8.1%	48.4%	4.8%
Tapi	Gujarat	28.4%	4.5%	56.7%	10.4%
Panchkula	Haryana	26.2%	3.1%	58.5%	12.3%
Ambala	Haryana	36.8%	10.3%	45.6%	7.4%
Yamunanagar	Haryana	46.7%	9.3%	34.7%	9.3%
Kurukshetra	Haryana	40.8%	5.1%	44.9%	9.2%
Kaithal	Haryana	43.0%	2.0%	51.0%	4.0%
Karnal	Haryana	40.4%	7.7%	46.2%	5.8%
Panipat	Haryana	42.0%	11.1%	42.0%	4.9%
Sonipat	Haryana	30.3%	17.1%	47.4%	5.3%
Jind	Haryana	45.1%	7.1%	38.1%	9.7%
Fatehabad	Haryana	45.5%	9.1%	39.7%	5.8%
Sirsa	Haryana	46.7%	1.7%	51.7%	0.0%
Hisar	Haryana	44.2%	8.1%	41.9%	5.8%
Bhiwani	Haryana	42.6%	3.7%	50.9%	2.8%
Rohtak	Haryana	40.2%	5.1%	42.7%	12.0%
Jhajjar	Haryana	47.1%	2.3%	34.5%	16.1%
Mahendragarh	Haryana	40.4%	16.0%	27.7%	16.0%
Rewari	Haryana	50.6%	6.0%	36.1%	7.2%
Gurgaon	Haryana	40.6%	13.0%	42.0%	4.3%
Mewat	Haryana	29.0%	2.9%	63.8%	4.3%
Faridabad	Haryana	31.0%	19.7%	31.0%	18.3%
Palwal	Haryana	40.7%	4.4%	46.9%	8.0%
Chamba	Himachal Pradesh	44.2%	10.5%	39.5%	5.8%
Kangra	Himachal Pradesh	45.5%	3.6%	30.9%	20.0%
Lahul and Spiti	Himachal Pradesh	55.9%	11.8%	23.5%	8.8%
Kullu	Himachal Pradesh	72.0%	2.7%	22.7%	2.7%
Mandi	Himachal Pradesh	50.0%	10.5%	25.0%	14.5%
Hamirpur	Himachal Pradesh	46.3%	13.4%	22.4%	17.9%
Una	Himachal Pradesh	53.2%	6.3%	21.5%	19.0%
Bilaspur	Himachal Pradesh	49.1%	8.8%	29.8%	12.3%
Solan	Himachal Pradesh	41.5%	7.7%	44.6%	6.2%
Sirmaur	Himachal Pradesh	48.8%	11.9%	36.9%	2.4%
Shimla	Himachal Pradesh	46.2%	5.8%	26.9%	21.2%
Kinnaur	Himachal Pradesh	60.9%	8.7%	28.3%	2.2%
Kupwara	Jammu and Kashmir	35.2%	16.4%	16.4%	32.0%
Badgam	Jammu and Kashmir	52.7%	5.5%	13.6%	28.2%
Leh	Jammu and Kashmir	42.3%	9.0%	21.8%	26.9%
Kargil	Jammu and Kashmir	33.0%	22.0%	21.1%	23.9%
Punch	Jammu and Kashmir	23.1%	20.8%	30.0%	26.2%
Rajouri	Jammu and Kashmir	32.1%	25.5%	19.8%	22.6%
Kathua	Jammu and Kashmir	31.7%	19.8%	23.8%	24.8%
Baramula	Jammu and Kashmir	30.4%	21.5%	25.3%	22.8%
Bandipore	Jammu and Kashmir	44.2%	11.5%	15.4%	28.8%
Srinagar	Jammu and Kashmir	30.0%	22.0%	34.0%	14.0%
Ganderbal	Jammu and Kashmir	41.0%	7.4%	10.7%	41.0%
Pulwama	Jammu and Kashmir	44.9%	18.0%	22.5%	14.6%

Shupiyan	Jammu and Kashmir	54.0%	11.0%	13.0%	22.0%
Anantnag	Jammu and Kashmir	54.3%	6.5%	9.8%	29.3%
Kulgam	Jammu and Kashmir	46.1%	14.7%	9.8%	29.4%
Doda	Jammu and Kashmir	29.9%	20.9%	17.9%	31.3%
Ramban	Jammu and Kashmir	26.1%	22.7%	11.8%	39.5%
Kishtwar	Jammu and Kashmir	28.8%	14.4%	19.8%	36.9%
Udhampur	Jammu and Kashmir	33.6%	15.9%	37.4%	13.1%
Reasi	Jammu and Kashmir	31.6%	19.7%	24.8%	23.9%
Jammu	Jammu and Kashmir	30.6%	16.1%	24.2%	29.0%
Samba	Jammu and Kashmir	40.4%	11.2%	9.0%	39.3%
Garhwa	Jharkhand	27.3%	10.6%	56.5%	5.6%
Chatra	Jharkhand	26.8%	14.2%	55.2%	3.8%
Kodarma	Jharkhand	37.0%	9.4%	49.3%	4.3%
Giridih	Jharkhand	38.5%	3.5%	58.0%	0.0%
Deoghar	Jharkhand	33.8%	11.0%	50.6%	4.5%
Godda	Jharkhand	29.1%	5.1%	63.2%	2.6%
Sahibganj	Jharkhand	21.2%	10.6%	63.7%	4.4%
Pakur	Jharkhand	25.0%	7.6%	62.1%	5.3%
Dhanbad	Jharkhand	30.5%	6.3%	57.5%	5.7%
Bokaro	Jharkhand	28.5%	5.0%	62.0%	4.5%
Lohardaga	Jharkhand	32.4%	13.0%	49.1%	5.6%
Purbi Singhbhum	Jharkhand	19.9%	13.0%	60.3%	6.8%
Palamu	Jharkhand	29.6%	7.7%	56.3%	6.3%
Latehar	Jharkhand	29.6%	9.3%	55.6%	5.6%
Hazaribagh	Jharkhand	29.4%	4.4%	61.8%	4.4%
Ramgarh	Jharkhand	34.4%	7.8%	54.4%	3.3%
Dumka	Jharkhand	17.6%	16.5%	57.6%	8.2%
Jamtara	Jharkhand	26.5%	8.8%	59.2%	5.4%
Ranchi	Jharkhand	35.8%	8.2%	51.5%	4.5%
Khunti	Jharkhand	25.0%	12.0%	54.3%	8.7%
Gumla	Jharkhand	17.8%	16.8%	57.9%	7.5%
Simdega	Jharkhand	29.3%	22.2%	45.5%	3.0%
Pashchimi Singhbhum	Jharkhand	14.9%	4.0%	78.2%	3.0%
Saraikela Kharsawan	Jharkhand	30.5%	10.2%	51.7%	7.6%
Belgaum	Karnataka	38.0%	7.6%	53.3%	1.1%
Bagalkot	Karnataka	27.8%	15.6%	52.2%	4.4%
Bijapur	Karnataka	30.1%	9.6%	57.5%	2.7%
Bidar	Karnataka	31.7%	9.8%	52.4%	6.1%
Raichur	Karnataka	37.3%	7.2%	53.0%	2.4%
Koppal	Karnataka	31.0%	8.0%	55.2%	5.7%
Gadag	Karnataka	29.7%	16.2%	48.6%	5.4%
Dharwad	Karnataka	37.8%	5.4%	52.7%	4.1%
Uttara Kannada	Karnataka	24.5%	20.4%	42.9%	12.2%
Haveri	Karnataka	37.3%	6.0%	49.3%	7.5%
Bellary	Karnataka	25.7%	13.9%	57.4%	3.0%
Chitradurga	Karnataka	23.4%	14.9%	51.1%	10.6%
Davanagere	Karnataka	26.8%	11.0%	54.9%	7.3%
Shimoga	Karnataka	43.8%	10.4%	33.3%	12.5%
Udupi	Karnataka	34.0%	17.0%	27.7%	21.3%
Chikmagalur	Karnataka	46.5%	20.9%	27.9%	4.7%

Tumkur	Karnataka	35.3%	7.8%	47.1%	9.8%
Bangalore	Karnataka	30.2%	9.4%	49.1%	11.3%
Mandya	Karnataka	48.1%	3.7%	31.5%	16.7%
Hassan	Karnataka	50.0%	10.4%	33.3%	6.3%
Dakshina Kannada	Karnataka	39.3%	10.7%	21.4%	28.6%
Kodagu	Karnataka	47.7%	9.2%	30.8%	12.3%
Mysore	Karnataka	40.3%	11.3%	32.3%	16.1%
Chamarajanagar	Karnataka	48.0%	16.0%	22.0%	14.0%
Gulbarga	Karnataka	19.8%	8.9%	65.3%	5.9%
Yadgir	Karnataka	26.5%	9.2%	60.2%	4.1%
Kolar	Karnataka	42.2%	9.4%	40.6%	7.8%
Chikkaballapura	Karnataka	45.7%	2.2%	47.8%	4.3%
Bangalore Rural	Karnataka	30.5%	8.5%	49.2%	11.9%
Ramanagara	Karnataka	38.0%	6.0%	40.0%	16.0%
Kasaragod	Kerala	40.7%	9.3%	25.9%	24.1%
Kannur	Kerala	34.0%	17.0%	25.5%	23.4%
Wayanad	Kerala	25.0%	16.7%	40.0%	18.3%
Kozhikode	Kerala	43.8%	22.9%	20.8%	12.5%
Malappuram	Kerala	26.7%	23.3%	28.3%	21.7%
Palakkad	Kerala	52.6%	12.3%	22.8%	12.3%
Thrissur	Kerala	34.2%	7.9%	28.9%	28.9%
Ernakulam	Kerala	45.0%	2.5%	27.5%	25.0%
Idukki	Kerala	42.4%	12.1%	24.2%	21.2%
Kottayam	Kerala	33.3%	12.5%	31.3%	22.9%
Alappuzha	Kerala	55.6%	11.1%	22.2%	11.1%
Pathanamthitta	Kerala	50.0%	2.9%	20.6%	26.5%
Kollam	Kerala	40.0%	22.9%	14.3%	22.9%
Thiruvananthapuram	Kerala	32.6%	21.7%	15.2%	30.4%
Lakshadweep	Lakshadweep	44.4%	13.6%	25.9%	16.0%
Sheopur	Madhya Pradesh	35.6%	3.0%	60.0%	1.5%
Morena	Madhya Pradesh	29.5%	6.1%	62.1%	2.3%
Bhind	Madhya Pradesh	31.9%	3.5%	61.1%	3.5%
Gwalior	Madhya Pradesh	38.6%	2.2%	57.0%	2.2%
Datia	Madhya Pradesh	28.9%	4.1%	65.3%	1.7%
Shivpuri	Madhya Pradesh	23.2%	10.1%	63.8%	2.9%
Tikamgarh	Madhya Pradesh	30.8%	7.7%	57.1%	4.4%
Chhatarpur	Madhya Pradesh	38.3%	7.5%	49.2%	5.0%
Panna	Madhya Pradesh	30.7%	10.2%	51.1%	8.0%
Sagar	Madhya Pradesh	40.0%	6.3%	46.3%	7.4%
Damoh	Madhya Pradesh	28.4%	12.2%	59.5%	0.0%
Satna	Madhya Pradesh	27.8%	6.7%	60.0%	5.6%
Rewa	Madhya Pradesh	39.0%	2.0%	51.0%	8.0%
Umaria	Madhya Pradesh	31.1%	12.6%	51.3%	5.0%
Neemuch	Madhya Pradesh	38.6%	8.9%	40.6%	11.9%
Mandsaur	Madhya Pradesh	50.0%	0.0%	44.5%	5.5%
Ratlam	Madhya Pradesh	26.5%	7.8%	52.0%	13.7%
Ujjain	Madhya Pradesh	37.9%	10.3%	40.5%	11.2%
Shajapur	Madhya Pradesh	23.0%	9.0%	66.0%	2.0%
Dewas	Madhya Pradesh	29.4%	9.5%	53.2%	7.9%
Dhar	Madhya Pradesh	30.2%	12.7%	50.8%	6.3%

Indore	Madhya Pradesh	36.8%	10.8%	43.1%	9.3%
Khargone (West Nimar)	Madhya Pradesh	34.1%	7.0%	51.9%	7.0%
Barwani	Madhya Pradesh	23.6%	12.0%	59.2%	5.2%
Rajgarh	Madhya Pradesh	35.3%	3.4%	59.5%	1.7%
Vidisha	Madhya Pradesh	41.1%	6.7%	50.0%	2.2%
Bhopal	Madhya Pradesh	28.9%	16.5%	45.4%	9.3%
Sehore	Madhya Pradesh	42.9%	5.7%	49.5%	1.9%
Raisen	Madhya Pradesh	35.0%	3.6%	59.1%	2.2%
Betul	Madhya Pradesh	34.0%	12.0%	48.0%	6.0%
Harda	Madhya Pradesh	38.8%	7.4%	52.1%	1.7%
Hoshangabad	Madhya Pradesh	35.6%	3.1%	58.2%	3.1%
Katni	Madhya Pradesh	18.3%	17.2%	53.8%	10.8%
Jabalpur	Madhya Pradesh	33.0%	6.6%	54.8%	5.6%
Narsimhapur	Madhya Pradesh	33.0%	17.6%	42.9%	6.6%
Dindori	Madhya Pradesh	31.2%	1.8%	65.1%	1.8%
Mandla	Madhya Pradesh	32.3%	6.3%	60.4%	1.0%
Chhindwara	Madhya Pradesh	33.0%	15.5%	43.3%	8.2%
Seoni	Madhya Pradesh	26.4%	13.2%	49.5%	11.0%
Balaghat	Madhya Pradesh	25.7%	11.9%	54.5%	7.9%
Guna	Madhya Pradesh	28.4%	3.9%	65.2%	2.6%
Ashoknagar	Madhya Pradesh	36.4%	5.5%	54.5%	3.6%
Shahdol	Madhya Pradesh	27.1%	10.6%	57.6%	4.7%
Anuppur	Madhya Pradesh	21.0%	9.0%	57.0%	13.0%
Sidhi	Madhya Pradesh	27.1%	6.2%	63.6%	3.1%
Singrauli	Madhya Pradesh	26.5%	8.8%	54.4%	10.2%
Jhabua	Madhya Pradesh	29.4%	9.5%	57.1%	4.0%
Alirajpur	Madhya Pradesh	28.3%	7.3%	62.3%	2.1%
Khandwa (East Nimar)	Madhya Pradesh	40.2%	4.5%	52.3%	3.0%
Burhanpur	Madhya Pradesh	35.5%	6.5%	52.7%	5.3%
Nandurbar	Maharashtra	29.9%	10.4%	54.5%	5.2%
Dhule	Maharashtra	26.6%	20.3%	44.3%	8.9%
Jalgaon	Maharashtra	32.1%	7.1%	57.1%	3.6%
Buldana	Maharashtra	39.4%	5.6%	52.1%	2.8%
Akola	Maharashtra	43.4%	9.6%	45.8%	1.2%
Washim	Maharashtra	33.9%	16.1%	40.3%	9.7%
Amravati	Maharashtra	36.7%	10.0%	50.0%	3.3%
Wardha	Maharashtra	32.6%	4.3%	52.2%	10.9%
Nagpur	Maharashtra	44.0%	2.7%	45.3%	8.0%
Bhandara	Maharashtra	56.6%	1.9%	39.6%	1.9%
Gondiya	Maharashtra	27.7%	7.7%	60.0%	4.6%
Gadchiroli	Maharashtra	34.0%	12.8%	44.7%	8.5%
Chandrapur	Maharashtra	42.4%	0.0%	50.8%	6.8%
Yavatmal	Maharashtra	16.0%	35.8%	42.0%	6.2%
Nanded	Maharashtra	39.8%	3.9%	55.3%	1.0%
Hingoli	Maharashtra	33.3%	7.4%	48.1%	11.1%
Parbhani	Maharashtra	34.8%	4.3%	58.7%	2.2%
Jalna	Maharashtra	32.9%	12.2%	53.7%	1.2%
Aurangabad	Maharashtra	47.1%	5.7%	41.4%	5.7%
Nashik	Maharashtra	29.8%	16.0%	51.1%	3.2%
Thane	Maharashtra	29.2%	13.9%	48.6%	8.3%

Mumbai Suburban	Maharashtra	50.0%	14.7%	20.6%	14.7%
Mumbai	Maharashtra	38.7%	12.9%	41.9%	6.5%
Raigarh	Maharashtra	32.3%	16.9%	38.5%	12.3%
Pune	Maharashtra	40.6%	10.9%	29.7%	18.8%
Ahmadnagar	Maharashtra	40.7%	7.4%	43.2%	8.6%
Bid	Maharashtra	31.2%	19.5%	35.1%	14.3%
Latur	Maharashtra	45.2%	5.6%	43.5%	5.6%
Osmanabad	Maharashtra	31.6%	11.8%	50.0%	6.6%
Solapur	Maharashtra	41.7%	14.3%	32.1%	11.9%
Satara	Maharashtra	47.7%	3.1%	35.4%	13.8%
Ratnagiri	Maharashtra	47.1%	0.0%	41.2%	11.8%
Sindhudurg	Maharashtra	32.1%	14.3%	28.6%	25.0%
Kolhapur	Maharashtra	52.2%	6.5%	37.0%	4.3%
Sangli	Maharashtra	43.9%	9.1%	34.8%	12.1%
Senapati (Excluding 3 Sub-Divisions)	Manipur	46.5%	8.2%	32.7%	12.6%
Tamenglong	Manipur	39.9%	12.1%	27.2%	20.8%
Churachandpur	Manipur	42.2%	9.5%	27.6%	20.7%
Bishnupur	Manipur	49.6%	11.8%	16.7%	22.0%
Thoubal	Manipur	44.2%	11.5%	23.1%	21.2%
Imphal West	Manipur	34.7%	13.3%	10.7%	41.3%
Imphal East	Manipur	47.7%	7.9%	20.6%	23.8%
Ukhrul	Manipur	47.6%	11.3%	21.8%	19.4%
Chandel	Manipur	43.7%	8.1%	34.1%	14.1%
West Garo Hills	Meghalaya	27.1%	26.0%	28.1%	18.8%
East Garo Hills	Meghalaya	19.2%	31.5%	12.3%	36.9%
South Garo Hills	Meghalaya	2.5%	65.0%	12.5%	20.0%
West Khasi Hills	Meghalaya	26.9%	25.0%	34.4%	13.7%
Ribhoi	Meghalaya	24.0%	28.6%	28.6%	18.8%
East Khasi Hills	Meghalaya	30.6%	23.4%	23.0%	23.0%
Jaintia Hills	Meghalaya	29.3%	18.7%	35.6%	16.4%
Mamit	Mizoram	42.5%	10.4%	21.7%	25.5%
Kolasib	Mizoram	39.6%	16.5%	13.7%	30.2%
Aizawl	Mizoram	45.6%	6.7%	7.8%	40.0%
Champhai	Mizoram	45.5%	9.7%	17.1%	27.6%
Serchhip	Mizoram	40.4%	14.6%	10.7%	34.3%
Lunglei	Mizoram	50.0%	8.9%	13.7%	27.4%
Lawngtlai	Mizoram	47.9%	12.8%	28.7%	10.6%
Saiha	Mizoram	38.3%	12.3%	31.7%	17.7%
Mon	Nagaland	40.2%	12.8%	28.2%	18.8%
Mokokchung	Nagaland	69.8%	11.6%	9.3%	9.3%
Zunheboto	Nagaland	42.9%	11.7%	19.5%	26.0%
Wokha	Nagaland	37.3%	11.9%	18.6%	32.2%
Dimapur	Nagaland	43.7%	9.5%	16.3%	30.5%
Phek	Nagaland	44.9%	12.1%	16.8%	26.2%
Tuensang	Nagaland	60.6%	4.8%	28.8%	5.8%
Longleng	Nagaland	43.8%	5.6%	32.6%	18.0%
Kiphire	Nagaland	29.4%	24.8%	22.0%	23.9%
Kohima	Nagaland	33.6%	16.1%	20.3%	30.1%
Peren	Nagaland	54.5%	6.6%	20.7%	18.2%
North West	Delhi	47.1%	8.8%	29.4%	14.7%

North	Delhi	47.8%	2.2%	43.5%	6.5%
North East	Delhi	47.9%	8.3%	29.2%	14.6%
East	Delhi	44.2%	9.3%	37.2%	9.3%
New Delhi	Delhi	42.9%	14.3%	23.8%	19.0%
Central	Delhi	40.0%	11.4%	42.9%	5.7%
West	Delhi	45.2%	3.2%	45.2%	6.5%
South West	Delhi	44.2%	9.6%	36.5%	9.6%
South	Delhi	42.5%	12.5%	35.0%	10.0%
Bargarh	Odisha	25.8%	13.5%	48.3%	12.4%
Jharsuguda	Odisha	36.0%	16.7%	40.0%	7.3%
Sambalpur	Odisha	34.7%	11.1%	47.2%	6.9%
Debagarh	Odisha	37.2%	9.0%	42.3%	11.5%
Sundargarh	Odisha	27.4%	10.8%	54.1%	7.6%
Kendujhar	Odisha	26.3%	22.1%	38.9%	12.6%
Mayurbhanj	Odisha	32.5%	12.5%	43.8%	11.3%
Baleshwar	Odisha	41.1%	4.1%	41.1%	13.7%
Bhadrak	Odisha	32.0%	13.4%	43.3%	11.3%
Kendrapara	Odisha	48.5%	4.1%	30.9%	16.5%
Jagatsinghapur	Odisha	46.1%	1.3%	28.9%	23.7%
Cuttack	Odisha	56.9%	6.9%	24.1%	12.1%
Jajapur	Odisha	37.5%	12.5%	26.3%	23.8%
Dhenkanal	Odisha	51.2%	6.1%	36.6%	6.1%
Anugul	Odisha	40.2%	8.8%	43.1%	7.8%
Nayagarh	Odisha	37.3%	14.7%	29.3%	18.7%
Khordha	Odisha	45.8%	13.0%	27.5%	13.7%
Puri	Odisha	56.6%	5.3%	18.4%	19.7%
Ganjam	Odisha	43.8%	7.5%	38.8%	10.0%
Gajapati	Odisha	41.9%	3.5%	46.5%	8.1%
Kandhamal	Odisha	34.5%	10.6%	47.8%	7.1%
Baudh	Odisha	31.9%	9.6%	55.3%	3.2%
Subarnapur	Odisha	26.1%	27.2%	38.0%	8.7%
Balangir	Odisha	32.3%	7.5%	57.0%	3.2%
Nuapada	Odisha	43.6%	6.0%	47.9%	2.6%
Kalahandi	Odisha	38.0%	10.1%	46.8%	5.1%
Rayagada	Odisha	24.3%	16.5%	52.4%	6.8%
Nabarangapur	Odisha	34.4%	9.9%	52.7%	3.1%
Koraput	Odisha	37.9%	5.8%	52.4%	3.9%
Malkangiri	Odisha	24.3%	18.8%	47.2%	9.7%
Yanam	Puducherry	27.5%	27.5%	21.7%	23.2%
Puducherry	Puducherry	23.1%	24.6%	18.5%	33.8%
Mahe	Puducherry	30.8%	23.1%	25.0%	21.2%
Karaikal	Puducherry	28.4%	20.3%	14.9%	36.5%
Gurdaspur	Punjab	64.9%	0.0%	31.2%	3.9%
Kapurthala	Punjab	50.8%	4.6%	35.4%	9.2%
Jalandhar	Punjab	42.9%	10.0%	38.6%	8.6%
Hoshiarpur	Punjab	46.0%	1.6%	44.4%	7.9%
Sangrur	Punjab	55.1%	1.4%	27.5%	15.9%
Fatehgarh Sahib	Punjab	55.9%	5.9%	29.4%	8.8%
Ludhiana	Punjab	51.9%	1.9%	40.4%	5.8%
Moga	Punjab	55.6%	6.3%	34.9%	3.2%

Firozpur	Punjab	45.6%	3.8%	40.5%	10.1%
Muktsar	Punjab	47.1%	4.3%	40.0%	8.6%
Faridkot	Punjab	44.8%	13.4%	34.3%	7.5%
Bathinda	Punjab	56.5%	11.6%	21.7%	10.1%
Mansa	Punjab	44.9%	2.6%	39.7%	12.8%
Patiala	Punjab	41.6%	11.2%	30.3%	16.9%
Amritsar	Punjab	53.9%	2.6%	35.5%	7.9%
Tarn Taran	Punjab	57.3%	4.0%	37.3%	1.3%
Rupnagar	Punjab	54.2%	4.2%	34.7%	6.9%
Sahibzada Ajit Singh Nagar	Punjab	65.0%	0.0%	26.3%	8.8%
Shahid Bhagat Singh Nagar	Punjab	46.1%	4.5%	38.2%	11.2%
Barnala	Punjab	39.3%	11.5%	27.9%	21.3%
Ganganagar	Rajasthan	50.4%	3.5%	45.2%	0.9%
Hanumangarh	Rajasthan	49.5%	3.0%	46.5%	1.0%
Bikaner	Rajasthan	42.8%	4.4%	51.6%	1.2%
Churu	Rajasthan	43.1%	11.0%	39.4%	6.4%
Jhunjhunun	Rajasthan	53.3%	1.0%	41.0%	4.8%
Alwar	Rajasthan	35.8%	5.8%	52.5%	5.8%
Bharatpur	Rajasthan	41.3%	5.8%	48.4%	4.5%
Dhaulpur	Rajasthan	33.3%	6.2%	58.0%	2.5%
Karauli	Rajasthan	46.0%	2.9%	48.2%	2.9%
Sawai Madhopur	Rajasthan	44.6%	5.4%	46.9%	3.1%
Dausa	Rajasthan	44.9%	5.1%	42.4%	7.6%
Jaipur	Rajasthan	47.4%	9.6%	36.4%	6.7%
Sikar	Rajasthan	49.1%	5.7%	37.7%	7.5%
Nagaur	Rajasthan	58.9%	4.7%	33.6%	2.8%
Jodhpur	Rajasthan	43.4%	3.0%	49.0%	4.5%
Jaisalmer	Rajasthan	32.9%	2.1%	61.0%	4.1%
Barmer	Rajasthan	41.8%	2.7%	54.1%	1.4%
Jalor	Rajasthan	34.1%	1.5%	64.4%	0.0%
Sirohi	Rajasthan	31.5%	3.5%	62.2%	2.8%
Pali	Rajasthan	39.2%	4.9%	52.9%	2.9%
Ajmer	Rajasthan	41.3%	3.9%	51.9%	2.9%
Tonk	Rajasthan	46.2%	4.3%	47.0%	2.6%
Bundi	Rajasthan	38.1%	1.9%	58.1%	1.9%
Bhilwara	Rajasthan	46.1%	5.9%	47.1%	1.0%
Rajsamand	Rajasthan	35.4%	6.3%	56.7%	1.6%
Dungarpur	Rajasthan	23.6%	1.6%	74.0%	0.8%
Banswara	Rajasthan	26.7%	0.8%	71.0%	1.5%
Chittaurgarh	Rajasthan	37.7%	1.4%	59.4%	1.4%
Kota	Rajasthan	38.7%	5.0%	53.0%	3.3%
Baran	Rajasthan	38.3%	2.6%	57.4%	1.7%
Jhalawar	Rajasthan	45.0%	0.0%	53.8%	1.3%
Udaipur	Rajasthan	30.4%	3.2%	64.0%	2.4%
Pratapgarh	Rajasthan	23.5%	1.7%	73.9%	0.8%
North District	Sikkim	29.0%	20.3%	15.9%	34.8%
West District	Sikkim	35.1%	22.8%	24.6%	17.5%
South District	Sikkim	31.0%	25.9%	20.7%	22.4%
East District	Sikkim	31.8%	18.7%	15.0%	34.6%

Thiruvallur	Tamil Nadu	29.8%	24.6%	21.1%	24.6%
Chennai	Tamil Nadu	14.3%	11.9%	40.5%	33.3%
Kancheepuram	Tamil Nadu	26.8%	26.8%	16.1%	30.4%
Vellore	Tamil Nadu	15.4%	28.2%	28.2%	28.2%
Tiruvannamalai	Tamil Nadu	13.4%	35.8%	26.9%	23.9%
Viluppuram	Tamil Nadu	20.2%	28.3%	26.3%	25.3%
Salem	Tamil Nadu	24.1%	16.9%	28.9%	30.1%
Namakkal	Tamil Nadu	19.2%	23.1%	19.2%	38.5%
Erode	Tamil Nadu	26.7%	26.7%	18.3%	28.3%
The Nilgiris	Tamil Nadu	22.2%	39.7%	20.6%	17.5%
Dindigul	Tamil Nadu	20.3%	31.9%	23.2%	24.6%
Karur	Tamil Nadu	17.7%	26.6%	24.1%	31.6%
Tiruchirappalli	Tamil Nadu	19.3%	21.1%	35.1%	24.6%
Perambalur	Tamil Nadu	16.2%	32.4%	17.6%	33.8%
Ariyalur	Tamil Nadu	25.9%	18.5%	31.5%	24.1%
Cuddalore	Tamil Nadu	30.4%	23.2%	30.4%	16.1%
Nagapattinam	Tamil Nadu	29.8%	36.8%	12.3%	21.1%
Thiruvarur	Tamil Nadu	22.4%	23.9%	28.4%	25.4%
Thanjavur	Tamil Nadu	20.3%	35.9%	18.8%	25.0%
Pudukkottai	Tamil Nadu	19.0%	29.8%	26.2%	25.0%
Sivaganga	Tamil Nadu	27.9%	16.4%	11.5%	44.3%
Madurai	Tamil Nadu	31.3%	20.8%	20.8%	27.1%
Theni	Tamil Nadu	31.5%	22.2%	20.4%	25.9%
Virudhunagar	Tamil Nadu	18.0%	28.0%	24.0%	30.0%
Ramanathapuram	Tamil Nadu	19.4%	14.5%	19.4%	46.8%
Thoothukkudi	Tamil Nadu	31.9%	25.5%	17.0%	25.5%
Tirunelveli	Tamil Nadu	18.4%	24.5%	16.3%	40.8%
Kanniyakumari	Tamil Nadu	20.6%	16.2%	13.2%	50.0%
Dharmapuri	Tamil Nadu	23.3%	20.5%	35.6%	20.5%
Krishnagiri	Tamil Nadu	21.6%	22.7%	29.5%	26.1%
Coimbatore	Tamil Nadu	27.4%	19.4%	19.4%	33.9%
Tiruppur	Tamil Nadu	26.5%	20.6%	27.9%	25.0%
West Tripura	Tripura	51.6%	6.3%	24.6%	17.5%
South Tripura	Tripura	50.9%	7.3%	38.2%	3.6%
Dhalai	Tripura	43.9%	9.1%	40.9%	6.1%
North Tripura	Tripura	63.4%	0.0%	32.3%	4.3%
Saharanpur	Uttar Pradesh	52.3%	3.5%	40.4%	3.8%
Muzaffarnagar	Uttar Pradesh	39.3%	5.1%	50.0%	5.6%
Bijnor	Uttar Pradesh	35.5%	2.1%	56.7%	5.7%
Moradabad	Uttar Pradesh	37.5%	5.5%	51.1%	5.9%
Rampur	Uttar Pradesh	38.4%	4.9%	53.7%	3.0%
Jyotiba Phule Nagar	Uttar Pradesh	32.8%	4.4%	61.3%	1.5%
Meerut	Uttar Pradesh	41.4%	3.7%	49.1%	5.7%
Baghpat	Uttar Pradesh	47.6%	2.4%	47.6%	2.4%
Ghaziabad	Uttar Pradesh	53.2%	1.5%	42.6%	2.7%
Gautam Buddha Nagar	Uttar Pradesh	50.5%	4.7%	37.8%	7.0%
Bulandshahr	Uttar Pradesh	37.7%	4.8%	54.1%	3.4%
Aligarh	Uttar Pradesh	34.5%	3.0%	58.9%	3.6%
Mahamaya Nagar	Uttar Pradesh	42.9%	6.1%	43.5%	7.5%
Mathura	Uttar Pradesh	46.0%	8.0%	38.7%	7.3%

Agra	Uttar Pradesh	41.9%	4.0%	50.3%	3.7%
Firozabad	Uttar Pradesh	47.4%	3.3%	44.5%	4.7%
Mainpuri	Uttar Pradesh	38.3%	5.0%	50.8%	5.8%
Budaun	Uttar Pradesh	29.1%	2.8%	65.9%	2.2%
Bareilly	Uttar Pradesh	37.7%	4.0%	53.8%	4.5%
Pilibhit	Uttar Pradesh	33.6%	2.7%	62.4%	1.3%
Shahjahanpur	Uttar Pradesh	32.6%	7.4%	57.8%	2.2%
Kheri	Uttar Pradesh	28.7%	7.4%	61.0%	2.9%
Sitapur	Uttar Pradesh	32.3%	6.2%	59.2%	2.3%
Hardoi	Uttar Pradesh	32.4%	5.9%	55.9%	5.9%
Unnao	Uttar Pradesh	41.8%	2.5%	53.3%	2.5%
Lucknow	Uttar Pradesh	35.3%	7.8%	56.9%	0.0%
Rae Bareli	Uttar Pradesh	44.0%	4.3%	50.9%	0.9%
Farrukhabad	Uttar Pradesh	45.7%	4.3%	45.7%	4.3%
Kannauj	Uttar Pradesh	39.2%	8.8%	49.6%	2.4%
Etawah	Uttar Pradesh	37.7%	1.5%	56.9%	3.8%
Auraiya	Uttar Pradesh	31.0%	3.5%	61.9%	3.5%
Kanpur Dehat	Uttar Pradesh	38.2%	4.5%	52.7%	4.5%
Kanpur Nagar	Uttar Pradesh	35.8%	5.7%	54.1%	4.4%
Jalaun	Uttar Pradesh	24.3%	0.0%	74.3%	1.4%
Jhansi	Uttar Pradesh	35.0%	8.6%	47.9%	8.6%
Lalitpur	Uttar Pradesh	22.1%	7.4%	68.4%	2.1%
Hamirpur	Uttar Pradesh	38.9%	1.4%	56.9%	2.8%
Mahoba	Uttar Pradesh	28.7%	3.2%	64.9%	3.2%
Banda	Uttar Pradesh	40.8%	0.0%	56.6%	2.6%
Chitrakoot	Uttar Pradesh	21.4%	12.6%	61.0%	5.0%
Fatehpur	Uttar Pradesh	35.2%	4.5%	58.0%	2.3%
Pratapgarh	Uttar Pradesh	45.8%	2.1%	52.1%	0.0%
Kaushambi	Uttar Pradesh	31.9%	0.9%	67.2%	0.0%
Allahabad	Uttar Pradesh	26.0%	5.3%	62.6%	6.1%
Bara Banki	Uttar Pradesh	39.8%	4.9%	51.5%	3.9%
Faizabad	Uttar Pradesh	32.8%	1.6%	64.0%	1.6%
Ambedkar Nagar	Uttar Pradesh	36.5%	6.6%	54.0%	2.9%
Sultanpur	Uttar Pradesh	43.0%	4.1%	47.9%	5.0%
Bahraich	Uttar Pradesh	24.5%	6.1%	64.4%	4.9%
Shrawasti	Uttar Pradesh	31.7%	8.7%	54.0%	5.6%
Balrampur	Uttar Pradesh	28.2%	13.4%	54.9%	3.5%
Gonda	Uttar Pradesh	30.9%	5.8%	56.8%	6.5%
Siddharth Nagar	Uttar Pradesh	38.7%	3.9%	54.8%	2.6%
Basti	Uttar Pradesh	44.8%	3.5%	49.0%	2.8%
Sant Kabir Nagar	Uttar Pradesh	37.4%	4.5%	53.5%	4.5%
Mahrajganj	Uttar Pradesh	30.9%	8.8%	51.5%	8.8%
Gorakhpur	Uttar Pradesh	42.0%	6.9%	48.1%	3.1%
Kushinagar	Uttar Pradesh	38.9%	9.4%	41.6%	10.1%
Deoria	Uttar Pradesh	44.3%	6.1%	48.9%	0.8%
Azamgarh	Uttar Pradesh	45.0%	5.4%	46.5%	3.1%
Mau	Uttar Pradesh	42.8%	5.1%	45.7%	6.5%
Ballia	Uttar Pradesh	39.6%	8.3%	49.3%	2.8%
Jaunpur	Uttar Pradesh	34.6%	7.8%	54.9%	2.6%
Ghazipur	Uttar Pradesh	43.6%	3.8%	51.1%	1.5%

Chandauli	Uttar Pradesh	41.5%	6.9%	45.3%	6.3%
Varanasi	Uttar Pradesh	33.5%	5.0%	59.4%	2.1%
Sant Ravidas Nagar (Bhadohi)	Uttar Pradesh	28.3%	8.0%	57.8%	5.9%
Mirzapur	Uttar Pradesh	27.6%	9.9%	59.2%	3.3%
Sonbhadra	Uttar Pradesh	34.5%	4.9%	59.2%	1.4%
Etah	Uttar Pradesh	42.5%	2.6%	52.9%	2.0%
Kanshiram Nagar	Uttar Pradesh	33.9%	4.1%	59.6%	2.3%
Uttarkashi	Uttarakhand	29.6%	7.0%	60.6%	2.8%
Chamoli	Uttarakhand	47.1%	12.9%	35.3%	4.7%
Rudraprayag	Uttarakhand	40.5%	8.3%	40.5%	10.7%
Tehri Garhwal	Uttarakhand	30.6%	8.2%	55.3%	5.9%
Dehradun	Uttarakhand	34.7%	14.6%	43.1%	7.6%
Garhwal	Uttarakhand	51.6%	6.3%	39.1%	3.1%
Pithoragarh	Uttarakhand	32.8%	9.8%	39.3%	18.0%
Bageshwar	Uttarakhand	38.5%	9.0%	41.0%	11.5%
Almora	Uttarakhand	44.0%	9.9%	39.6%	6.6%
Champawat	Uttarakhand	32.5%	15.0%	41.3%	11.3%
Nainital	Uttarakhand	48.6%	7.4%	28.6%	15.4%
Udham Singh Nagar	Uttarakhand	42.9%	10.8%	35.5%	10.8%
Hardwar	Uttarakhand	46.1%	5.7%	41.2%	6.9%
Darjiling	West Bengal	47.5%	9.8%	24.6%	18.0%
Jalpaiguri	West Bengal	23.1%	13.8%	33.8%	29.2%
Koch Bihar	West Bengal	30.6%	16.1%	25.8%	27.4%
Uttar Dinajpur	West Bengal	35.6%	18.3%	34.6%	11.5%
Dakshin Dinajpur	West Bengal	36.7%	11.7%	30.0%	21.7%
Maldah	West Bengal	34.6%	8.4%	48.6%	8.4%
Murshidabad	West Bengal	29.7%	18.9%	39.6%	11.7%
Birbhum	West Bengal	29.5%	19.3%	39.8%	11.4%
Barddhaman	West Bengal	25.0%	23.7%	30.3%	21.1%
Nadia	West Bengal	31.4%	20.9%	16.3%	31.4%
North Twenty Four Parganas	West Bengal	47.3%	9.1%	16.4%	27.3%
Hugli	West Bengal	34.9%	19.0%	28.6%	17.5%
Bankura	West Bengal	27.0%	25.7%	31.1%	16.2%
Puruliya	West Bengal	24.6%	24.6%	43.4%	7.4%
Haora	West Bengal	33.9%	16.1%	29.0%	21.0%
Kolkata	West Bengal	41.3%	8.7%	30.4%	19.6%
South Twenty Four Parganas	West Bengal	26.1%	15.3%	31.5%	27.0%
Paschim Medinipur	West Bengal	28.4%	21.6%	32.4%	17.6%
Purba Medinipur	West Bengal	31.9%	16.7%	29.2%	22.2%
Adilabad	Telangana	38.3%	8.3%	48.3%	5.0%
Nizamabad	Telangana	50.0%	12.5%	32.5%	5.0%
Karimnagar	Telangana	52.8%	11.3%	22.6%	13.2%
Medak	Telangana	37.1%	9.7%	45.2%	8.1%
Hyderabad	Telangana	43.7%	9.9%	25.4%	21.1%
Rangareddy	Telangana	46.3%	7.4%	33.3%	13.0%
Mahbubnagar	Telangana	54.2%	3.6%	32.5%	9.6%
Nalgonda	Telangana	32.9%	8.6%	44.3%	14.3%
Warangal	Telangana	44.4%	5.6%	29.6%	20.4%
Khammam	Telangana	44.4%	5.6%	31.5%	18%
