# Supplementary material for: A typology of dietary and anthropometric measures of nutritional need among children across districts and parliamentary constituencies in India, 2016
Source: J Glob Health. 2020 Aug 24;10(2):020424. doi: 10.7189/jogh.10.020424 (PMC7569191; doi:10.7189/jogh.10.020424)
Supplement: Online Supplementary Document [file jogh-10-020424-s001.zip › Beckerman AU Supplementary Table 4.pdf]

Supplementary Table 4: Parliamentary Constituency (PC)-level percentage of types of Dietary and Anthropometric Failures by decile in India.

Paper Title: A Typology of Dietary and Anthropometric Measures of Nutritional Need Among Children Across Districts and Parliamentary Constituencies in India, 2016

Suggested citation: Beckerman-Hsu, JP, Chatterjee, P, Kim, R, Sharma, S, Subramanian SV. A Typology of Dietary and Anthropometric Measures of Nutritional Need Among Children Across Districts and Parliamentary Constituencies in India, 2016. *Journal of Global Health.* 2020. doi: 10.7189/jogh.10.020424

Table S4: Parliamentary Constituency (PC)-level percentage of types of Dietary and Anthropometric Failures by decile in India.

Deciles	1	2	3	4	5	6	7	8	9	10

PC	State	Diet Failure only (DFO)	Anthropometric Failure only (AFO)	Both Failures (BF)	Neither Failure (NF)
Andaman & Nicobar Islands	Andaman & Nicobar Island	44.2%	17.1%	22.7%	16.0%
Araku	Andhra Pradesh	41.7%	11.3%	35.2%	11.8%
Anakapalli	Andhra Pradesh	46.4%	13.9%	28.0%	11.6%
Srikakulam	Andhra Pradesh	48.3%	5.0%	36.7%	10.0%
Eluru	Andhra Pradesh	49.1%	7.8%	39.0%	4.1%
Rajahmundry	Andhra Pradesh	48.9%	6.8%	36.0%	8.3%
Narsapuram	Andhra Pradesh	50.0%	7.5%	40.0%	2.5%
Amlapuram	Andhra Pradesh	48.4%	6.5%	33.9%	11.3%
Narasaraopet	Andhra Pradesh	51.9%	5.6%	25.9%	16.7%
Machilipatnam	Andhra Pradesh	46.8%	8.5%	36.2%	8.5%
Guntur	Andhra Pradesh	51.8%	5.6%	25.9%	16.7%
Ongole	Andhra Pradesh	37.3%	14.7%	29.3%	18.7%
Bapatla	Andhra Pradesh	43.7%	10.7%	27.8%	17.8%
Kurnool	Andhra Pradesh	39.4%	9.1%	45.5%	6.1%
Vizianagaram	Andhra Pradesh	38.4%	9.6%	40.3%	11.7%
Kakinada	Andhra Pradesh	48.4%	6.5%	33.9%	11.3%
Rajampet	Andhra Pradesh	45.9%	7.9%	39.4%	6.7%
Nellore	Andhra Pradesh	37.9%	12.4%	42.0%	7.7%
Anantapur	Andhra Pradesh	30.7%	18.7%	40.0%	10.7%
Kadapa	Andhra Pradesh	32.8%	9.4%	43.8%	14.1%
Nandyal	Andhra Pradesh	39.4%	9.1%	45.5%	6.1%
Chittoor	Andhra Pradesh	52.8%	7.1%	37.1%	2.9%
Tirupati	Andhra Pradesh	44.4%	9.9%	41.0%	4.7%
Hindupur	Andhra Pradesh	30.7%	18.7%	40.0%	10.7%
Vijayawada	Andhra Pradesh	46.8%	8.5%	36.2%	8.5%
Visakhapatnam	Andhra Pradesh	44.2%	13.8%	30.2%	11.8%
Arunachal West	Arunanchal Pradesh	38.6%	12.8%	35.6%	13.0%
Arunachal East	Arunanchal Pradesh	32.9%	16.1%	24.4%	26.6%
Lakhimpur	Assam	38.7%	11.8%	30.1%	19.5%
Dibrugarh	Assam	32.4%	18.8%	33.3%	15.5%
Jorhat	Assam	39.5%	13.6%	26.1%	20.8%
Tezpur	Assam	39.2%	12.9%	38.5%	9.4%
Kaliabor	Assam	31.4%	17.9%	31.3%	19.3%
Mangaldoi	Assam	37.9%	13.1%	41.2%	7.8%
Nagaon	Assam	39.8%	11.5%	35.0%	13.6%
Autonomous District	Assam	40.7%	17.3%	26.7%	15.3%
Dhubri	Assam	29.8%	13.8%	46.0%	10.3%
Karimganj	Assam	37.2%	12.7%	39.6%	10.4%
Silchar	Assam	47.0%	3.6%	49.4%	0.0%
Kokrajhar	Assam	36.5%	15.4%	32.6%	15.5%
Guwahati	Assam	35.5%	11.4%	32.1%	21.1%
Barpeta	Assam	36.1%	14.8%	37.4%	11.7%

Muzaffarpur	Bihar	34.8%	5.7%	51.1%	8.5%
Valmiki Nagar	Bihar	37.6%	12.0%	41.4%	9.0%
Araria	Bihar	28.9%	16.4%	47.8%	7.0%
Gopalganj	Bihar	40.3%	12.2%	38.8%	8.6%
Siwan	Bihar	40.4%	8.6%	43.7%	7.3%
Vaishali	Bihar	34.9%	6.6%	50.5%	8.0%
Jhanjharpur	Bihar	35.5%	16.3%	43.6%	4.7%
Supaul	Bihar	30.3%	10.6%	54.0%	5.1%
Pashchim Champaran	Bihar	35.1%	9.4%	46.7%	8.8%
Madhubani	Bihar	34.9%	14.5%	44.4%	6.2%
Kishanganj	Bihar	29.1%	12.9%	51.5%	6.5%
Darbhanga	Bihar	34.0%	11.4%	45.9%	8.8%
Purnia	Bihar	34.0%	11.3%	48.9%	5.8%
Maharajganj	Bihar	37.0%	9.8%	44.2%	8.9%
Madhepura	Bihar	31.7%	6.4%	57.2%	4.7%
Begusarai	Bihar	37.1%	7.5%	49.5%	5.9%
Arrah	Bihar	27.7%	10.4%	54.3%	7.5%
Sasaram	Bihar	31.5%	3.6%	61.6%	3.3%
Nawada	Bihar	31.5%	14.2%	45.6%	8.6%
Banka	Bihar	29.4%	9.8%	56.2%	4.5%
Nalanda	Bihar	30.8%	11.9%	53.1%	4.2%
Katihar	Bihar	32.0%	7.7%	55.6%	4.7%
Samastipur	Bihar	28.3%	7.4%	56.0%	8.3%
Khagaria	Bihar	32.0%	8.9%	53.1%	6.0%
Pataliputra	Bihar	31.6%	4.8%	57.7%	5.8%
Buxar	Bihar	31.5%	6.9%	53.7%	7.9%
Patna Sahib	Bihar	31.7%	4.9%	57.5%	5.8%
Bhagalpur	Bihar	29.6%	12.8%	52.2%	5.4%
Munger	Bihar	30.7%	8.6%	55.6%	5.1%
Purba Champaran	Bihar	33.3%	7.4%	50.6%	8.6%
Sheohar	Bihar	31.2%	9.9%	52.5%	6.3%
Sitamarhi	Bihar	27.7%	12.5%	56.5%	3.3%
Ujiapur	Bihar	28.6%	7.4%	56.3%	7.7%
Hajipur	Bihar	35.7%	11.0%	47.8%	5.5%
Karakat	Bihar	29.4%	7.4%	59.6%	3.6%
Saran (Chhapra)	Bihar	35.4%	10.4%	44.4%	9.7%
Jamui	Bihar	26.1%	11.8%	57.7%	4.4%
Aurangabad	Bihar	27.2%	13.2%	56.9%	2.7%
Gaya	Bihar	27.3%	13.9%	57.1%	1.8%
Jahanabad	Bihar	27.0%	12.9%	54.9%	5.2%
Chandigarh	Chandigarh	56.3%	2.1%	39.6%	2.1%
Janjgir-Champa	Chhattisgarh	37.6%	13.0%	40.4%	8.9%
Raipur	Chhattisgarh	40.1%	7.8%	43.8%	8.3%
Surguja	Chhattisgarh	36.0%	13.5%	37.9%	12.6%
Bilaspur	Chhattisgarh	34.3%	12.4%	47.6%	5.7%
Rajnandgaon	Chhattisgarh	29.6%	9.7%	51.6%	9.1%
Durg	Chhattisgarh	46.2%	5.4%	43.5%	4.9%
Mahasamund	Chhattisgarh	35.7%	11.8%	44.1%	8.5%
Raigarh	Chhattisgarh	42.4%	10.9%	34.9%	11.9%
Kanker	Chhattisgarh	37.3%	6.4%	51.2%	5.1%

Bastar	Chhattisgarh	25.6%	8.1%	62.4%	4.0%
Korba	Chhattisgarh	35.6%	9.7%	47.1%	7.7%
Dadra & Nagar Haveli	Dadara & Nagar Havelli	33.5%	2.2%	63.4%	0.9%
Daman & Diu	Daman & Diu	47.6%	6.8%	30.0%	15.6%
South Goa	Goa	41.9%	3.6%	33.0%	21.5%
North Goa	Goa	51.9%	3.8%	30.8%	13.5%
Bardoli	Gujarat	34.8%	6.7%	51.5%	7.0%
Junagadh	Gujarat	42.6%	3.7%	50.2%	3.5%
Surat	Gujarat	38.7%	8.1%	48.4%	4.8%
Kheda	Gujarat	35.1%	6.9%	53.3%	4.6%
Ahmadabad (West)	Gujarat	35.6%	3.4%	54.2%	6.8%
Jamnagar	Gujarat	29.1%	10.1%	51.9%	8.9%
Sabar Kantha	Gujarat	42.7%	6.1%	48.8%	2.4%
Banas Kantha	Gujarat	35.0%	7.8%	50.5%	6.8%
Patan	Gujarat	41.8%	6.1%	47.8%	4.3%
Panch Mahals	Gujarat	30.4%	6.4%	59.2%	3.9%
Dohad	Gujarat	27.6%	7.9%	61.2%	3.3%
Vadodara	Gujarat	55.2%	12.1%	31.0%	1.7%
Anand	Gujarat	34.3%	4.3%	55.7%	5.7%
Amreli	Gujarat	40.5%	7.5%	45.3%	6.7%
Ahmadabad (East)	Gujarat	39.1%	3.2%	54.4%	3.3%
Rajkot	Gujarat	47.0%	4.6%	40.9%	7.6%
Surendranagar	Gujarat	32.8%	3.0%	60.7%	3.5%
Navsari	Gujarat	32.9%	8.4%	51.3%	7.4%
Bharuch	Gujarat	35.9%	7.6%	52.0%	4.5%
Chhota Udaipur	Gujarat	44.3%	9.2%	44.3%	2.2%
Porbandar	Gujarat	47.8%	4.0%	42.8%	5.5%
Valsad	Gujarat	35.1%	15.6%	42.6%	6.7%
Gandhinagar	Gujarat	37.7%	3.2%	54.4%	4.7%
Mahesana	Gujarat	33.9%	3.8%	57.4%	4.8%
Bhavnagar	Gujarat	35.5%	4.8%	53.2%	6.5%
Kachchh	Gujarat	33.7%	7.4%	53.3%	5.6%
Ambala	Haryana	38.3%	8.3%	44.1%	9.2%
Krukshetra	Haryana	42.9%	4.5%	45.8%	6.8%
Sirsa	Haryana	46.0%	5.1%	45.6%	3.3%
Karnal	Haryana	41.1%	9.2%	44.3%	5.4%
Sonipat	Haryana	34.8%	14.0%	44.6%	6.6%
Hisar	Haryana	44.1%	7.5%	42.3%	6.1%
Rohtak	Haryana	44.4%	4.1%	38.6%	12.9%
Bhiwani - Mahendragarh	Haryana	41.7%	8.7%	41.4%	8.2%
Gurgaon	Haryana	39.4%	8.8%	46.8%	4.9%
Faridabad	Haryana	34.1%	14.9%	36.0%	15.0%
Hamirpur	Himachal Pradesh	49.4%	9.0%	24.7%	16.9%
Kangra	Himachal Pradesh	45.2%	4.8%	32.4%	17.6%
Shimla	Himachal Pradesh	45.5%	8.4%	35.9%	10.1%
Mandi	Himachal Pradesh	54.6%	8.2%	26.9%	10.3%
Leh (Ladakh)	Jammu & Kashmir	36.7%	15.8%	20.4%	27.1%
Jammu	Jammu & Kashmir	31.2%	17.7%	22.3%	28.7%
Srinagar	Jammu & Kashmir	37.3%	14.8%	22.7%	25.2%
Anantnag	Jammu & Kashmir	50.5%	10.4%	12.8%	26.3%

Baramula	Jammu & Kashmir	34.9%	17.6%	19.9%	27.6%
Udhampur	Jammu & Kashmir	32.0%	17.4%	22.7%	27.9%
Jamshedpur	Jharkhand	19.9%	13.0%	60.3%	6.8%
Singhbhum	Jharkhand	19.2%	5.7%	70.9%	4.3%
Rajmahal	Jharkhand	23.5%	8.9%	62.9%	4.6%
Dumka	Jharkhand	24.3%	12.0%	57.4%	6.3%
Godda	Jharkhand	29.9%	9.2%	56.8%	4.1%
Palamu	Jharkhand	28.5%	9.0%	56.4%	6.0%
Hazaribagh	Jharkhand	31.7%	5.9%	58.4%	4.0%
Dhanbad	Jharkhand	29.7%	5.8%	59.3%	5.2%
Kodarma	Jharkhand	37.7%	5.2%	55.7%	1.4%
Lohardaga	Jharkhand	26.7%	13.3%	53.9%	6.1%
Khunti	Jharkhand	27.6%	14.5%	51.8%	6.0%
Chatra	Jharkhand	28.2%	11.4%	55.5%	4.8%
Ranchi	Jharkhand	34.9%	8.5%	51.5%	5.0%
Giridih	Jharkhand	31.3%	5.3%	59.3%	4.1%
Gulbarga	Karnataka	20.6%	8.9%	64.7%	5.7%
Bijapur	Karnataka	30.1%	9.6%	57.5%	2.7%
Chikkodi	Karnataka	38.0%	7.6%	53.3%	1.1%
Raichur	Karnataka	33.2%	8.0%	55.7%	3.0%
Koppal	Karnataka	31.8%	8.6%	55.0%	4.6%
Haveri	Karnataka	34.5%	9.8%	49.0%	6.7%
Davanagere	Karnataka	26.8%	11.0%	54.9%	7.3%
Chikkaballapura	Karnataka	36.4%	6.2%	48.4%	9.0%
Udupi Chikmagalur	Karnataka	39.8%	18.8%	27.8%	13.6%
Tumkur	Karnataka	35.3%	7.8%	47.1%	9.8%
Kolar	Karnataka	43.2%	7.3%	42.7%	6.8%
Bangalore Rural	Karnataka	34.0%	7.8%	45.0%	13.2%
Dharwad	Karnataka	37.8%	5.5%	52.2%	4.5%
Bangalore North	Karnataka	30.2%	9.4%	49.1%	11.3%
Dakshina Kannada	Karnataka	39.0%	11.0%	21.7%	28.2%
Mysore	Karnataka	42.2%	10.8%	31.9%	15.2%
Chamrajnagar	Karnataka	44.0%	13.5%	27.4%	15.1%
Mandya	Karnataka	47.2%	4.6%	31.6%	16.6%
Bellary	Karnataka	25.7%	13.9%	57.4%	3.0%
Chitradurga	Karnataka	26.8%	12.9%	49.9%	10.4%
Bidar	Karnataka	28.5%	9.5%	55.9%	6.1%
Uttara Kannada	Karnataka	28.4%	16.7%	45.9%	9.0%
Shimoga	Karnataka	43.0%	10.9%	32.9%	13.2%
Hassan	Karnataka	49.4%	12.2%	32.4%	6.0%
Belgaum	Karnataka	38.0%	7.7%	53.3%	1.1%
Bangalore South	Karnataka	30.2%	9.4%	49.1%	11.3%
Bangalore Central	Karnataka	30.2%	9.4%	49.1%	11.3%
Bagalkot	Karnataka	28.1%	15.6%	51.7%	4.6%
Malappuram	Kerala	26.7%	23.3%	28.3%	21.7%
Pathanamthitta	Kerala	45.7%	5.5%	23.3%	25.5%
Mavelikkara	Kerala	48.9%	14.6%	20.8%	15.7%
Thiruvananthapuram	Kerala	32.6%	21.7%	15.2%	30.4%
Palakkad	Kerala	52.6%	12.3%	22.8%	12.3%
Thrissur	Kerala	34.2%	7.9%	28.9%	28.9%

Alathur	Kerala	46.3%	10.8%	24.9%	18.0%
Kasaragod	Kerala	38.8%	11.6%	25.8%	23.9%
Attingal	Kerala	32.6%	21.7%	15.2%	30.4%
Vadakara	Kerala	42.2%	22.0%	21.6%	14.2%
Kozhikode	Kerala	43.8%	22.9%	20.8%	12.5%
Kannur	Kerala	34.0%	17.0%	25.5%	23.4%
Chalakudy	Kerala	41.4%	4.3%	28.0%	26.3%
Idukki	Kerala	43.1%	9.6%	25.1%	22.2%
Alappuzha	Kerala	54.4%	11.9%	21.7%	12.0%
Kottayam	Kerala	35.0%	11.1%	30.7%	23.2%
Kollam	Kerala	40.0%	22.9%	14.3%	22.9%
Ernakulam	Kerala	45.0%	2.5%	27.5%	25.0%
Wayanad	Kerala	29.4%	21.3%	30.3%	19.0%
Ponnani	Kerala	28.8%	22.4%	27.9%	20.9%
Lakshadweep	Lakshadweep	44.4%	13.6%	25.9%	16.0%
Bhind	Madhya Pradesh	31.9%	3.5%	61.7%	2.9%
Balaghat	Madhya Pradesh	25.9%	12.2%	53.2%	8.7%
Hoshangabad	Madhya Pradesh	34.6%	7.9%	53.4%	4.1%
Dhar	Madhya Pradesh	31.1%	12.4%	49.7%	6.8%
Indore	Madhya Pradesh	36.8%	10.8%	43.1%	9.3%
Gwalior	Madhya Pradesh	34.3%	4.4%	58.8%	2.4%
Sidhi	Madhya Pradesh	26.9%	7.9%	58.8%	6.5%
Rajgarh	Madhya Pradesh	31.8%	4.4%	61.8%	2.0%
Sagar	Madhya Pradesh	40.5%	6.5%	47.9%	5.2%
Damoh	Madhya Pradesh	34.1%	9.3%	53.1%	3.5%
Shahdol	Madhya Pradesh	24.6%	11.3%	55.1%	9.0%
Dewas	Madhya Pradesh	28.2%	8.8%	58.7%	4.4%
Ujjain	Madhya Pradesh	36.0%	9.9%	42.4%	11.6%
Bhopal	Madhya Pradesh	30.3%	15.4%	45.8%	8.5%
Vidisha	Madhya Pradesh	37.8%	5.5%	53.9%	2.8%
Ratlam	Madhya Pradesh	28.2%	8.3%	57.2%	6.3%
Rewa	Madhya Pradesh	38.8%	2.1%	51.1%	8.0%
Satna	Madhya Pradesh	27.8%	6.7%	60.0%	5.6%
Mandsaur	Madhya Pradesh	43.2%	4.0%	44.2%	8.6%
Guna	Madhya Pradesh	29.1%	6.9%	60.9%	3.1%
Chhindwara	Madhya Pradesh	33.0%	15.5%	43.3%	8.2%
Betul	Madhya Pradesh	36.0%	9.9%	49.6%	4.6%
Khargone	Madhya Pradesh	28.5%	9.7%	55.8%	6.0%
Jabalpur	Madhya Pradesh	33.0%	6.6%	54.8%	5.6%
Mandla	Madhya Pradesh	30.4%	8.5%	56.4%	4.7%
Morena	Madhya Pradesh	31.1%	5.3%	61.6%	2.1%
Tikamgarh	Madhya Pradesh	33.3%	7.6%	54.5%	4.6%
Khajuraho	Madhya Pradesh	29.9%	10.9%	51.6%	7.6%
Khandwa	Madhya Pradesh	36.1%	6.3%	52.4%	5.2%
Buldana	Maharashtra	39.4%	5.6%	52.1%	2.8%
Madha	Maharashtra	44.2%	9.6%	33.5%	12.7%
Satara	Maharashtra	47.7%	3.1%	35.4%	13.8%
Jalgaon	Maharashtra	32.1%	7.1%	57.1%	3.6%
Akola	Maharashtra	41.6%	10.8%	44.8%	2.8%
Sangli	Maharashtra	43.9%	9.1%	34.8%	12.1%

Solapur	Maharashtra	41.7%	14.3%	32.1%	11.9%
Amravati	Maharashtra	36.7%	10.0%	50.0%	3.3%
Ramtek	Maharashtra	44.0%	2.7%	45.3%	8.0%
Nandurbar	Maharashtra	28.7%	14.0%	50.8%	6.5%
Bhandara - Gondiya	Maharashtra	41.4%	4.9%	50.3%	3.3%
Wardha	Maharashtra	34.3%	6.7%	51.3%	7.8%
Shirur	Maharashtra	40.6%	10.9%	29.7%	18.8%
Beed	Maharashtra	31.2%	19.5%	35.1%	14.3%
Maval	Maharashtra	35.5%	14.6%	35.1%	14.8%
Parbhani	Maharashtra	34.2%	6.9%	57.0%	1.9%
Raigarh	Maharashtra	36.9%	11.7%	39.3%	12.1%
Osmanabad	Maharashtra	36.1%	11.1%	45.4%	7.4%
Hatkanangle	Maharashtra	49.9%	7.2%	36.4%	6.5%
Dhule	Maharashtra	28.2%	18.1%	47.7%	6.0%
Garhchiroli - Chimur	Maharashtra	35.6%	7.7%	49.6%	7.2%
Raver	Maharashtra	33.2%	6.9%	56.4%	3.5%
Biwandi	Maharashtra	29.2%	13.9%	48.6%	8.3%
Dindori	Maharashtra	29.8%	16.0%	51.1%	3.2%
Jalna	Maharashtra	41.4%	8.3%	46.4%	3.9%
Aurangabad	Maharashtra	47.1%	5.7%	41.4%	5.7%
Chandrapur	Maharashtra	32.3%	13.6%	47.5%	6.6%
Nashik	Maharashtra	29.8%	16.0%	51.1%	3.2%
Shirdi	Maharashtra	40.7%	7.4%	43.2%	8.6%
Hingoli	Maharashtra	33.6%	9.8%	50.2%	6.5%
Ahmadnagar	Maharashtra	40.7%	7.4%	43.2%	8.6%
Palghar	Maharashtra	29.2%	13.9%	48.6%	8.3%
Latur	Maharashtra	43.4%	6.1%	45.2%	5.3%
Baramati	Maharashtra	40.6%	10.9%	29.7%	18.8%
Ratnagiri - Sindhudurg	Maharashtra	39.9%	6.9%	35.1%	18.1%
Kolhapur	Maharashtra	52.2%	6.5%	37.0%	4.3%
Thane	Maharashtra	29.4%	13.9%	48.3%	8.4%
Mumbai North	Maharashtra	49.9%	14.7%	20.7%	14.7%
Mumbai North-West	Maharashtra	50.0%	14.7%	20.6%	14.7%
Mumbai North-East	Maharashtra	50.0%	14.7%	20.6%	14.7%
Mumbai North-Central	Maharashtra	49.9%	14.7%	20.7%	14.7%
Mumbai South	Maharashtra	38.7%	12.9%	41.9%	6.5%
Mumbai South-Central	Maharashtra	43.8%	13.7%	32.4%	10.1%
Kalyan	Maharashtra	29.2%	13.9%	48.6%	8.3%
Pune	Maharashtra	40.6%	10.9%	29.7%	18.8%
Nanded	Maharashtra	39.8%	3.9%	55.3%	1.0%
Yavatmal - Washim	Maharashtra	22.1%	29.1%	41.4%	7.4%
Nagpur	Maharashtra	44.0%	2.7%	45.3%	8.0%
Inner Manipur	Manipur	48.0%	8.7%	19.8%	23.5%
Outer Manipur	Manipur	44.2%	10.1%	27.1%	18.6%
Tura	Meghalaya	21.3%	33.1%	21.3%	24.2%
Shillong	Meghalaya	28.7%	23.4%	28.7%	19.2%
Mizoram	Mizoram	45.1%	9.8%	14.9%	30.3%
Nagaland	Nagaland	46.6%	11.2%	20.2%	22.0%
West Delhi	NCT of Delhi	44.7%	6.4%	40.9%	8.0%
North West Delhi	NCT of Delhi	46.6%	7.4%	33.4%	12.6%

Chandni Chowk	NCT of Delhi	46.5%	6.8%	35.5%	11.2%
North East Delhi	NCT of Delhi	47.6%	6.9%	33.1%	12.4%
South Delhi	NCT of Delhi	42.8%	12.1%	35.2%	9.9%
East Delhi	NCT of Delhi	43.7%	10.2%	36.6%	9.5%
New Delhi	NCT of Delhi	43.4%	10.9%	34.3%	11.4%
Bhadrak	Odisha	34.8%	10.6%	42.6%	12.1%
Jajapur	Odisha	37.5%	12.5%	26.3%	23.8%
Sambalpur	Odisha	37.0%	10.0%	45.1%	7.9%
Baleshwar	Odisha	40.1%	5.0%	41.4%	13.4%
Kendujhar	Odisha	27.2%	20.7%	39.6%	12.4%
Mayurbhanj	Odisha	32.5%	12.5%	43.8%	11.3%
Sundargarh	Odisha	27.4%	10.8%	54.1%	7.6%
Bargarh	Odisha	28.7%	14.4%	46.0%	10.9%
Dhenkanal	Odisha	47.7%	7.0%	38.6%	6.6%
Bolangir	Odisha	30.5%	13.0%	51.7%	4.8%
Kalahandi	Odisha	39.6%	9.0%	47.1%	4.4%
Kandhamal	Odisha	36.3%	10.5%	44.2%	9.0%
Kendrapara	Odisha	56.3%	6.7%	24.6%	12.4%
Cuttack	Odisha	55.0%	7.7%	24.6%	12.7%
Bhubaneswar	Odisha	46.6%	12.5%	27.2%	13.6%
Aska	Odisha	43.8%	7.5%	38.8%	10.0%
Jagatsinghpur	Odisha	50.7%	3.2%	25.0%	21.1%
Nabarangapur	Odisha	32.2%	11.6%	51.1%	5.0%
Koraput	Odisha	31.2%	11.1%	52.4%	5.3%
Puri	Odisha	50.1%	8.9%	22.7%	18.3%
Berhampur.	Odisha	43.3%	6.4%	40.8%	9.5%
Puducherry	Puducherry	24.1%	24.2%	18.2%	33.6%
Jalandhar	Punjab	42.9%	10.0%	38.6%	8.6%
Hoshiarpur	Punjab	49.7%	1.9%	40.7%	7.6%
Fatehgarh Sahib	Punjab	53.2%	3.2%	35.8%	7.8%
Firozpur	Punjab	45.9%	3.9%	40.4%	9.8%
Patiala	Punjab	46.0%	8.5%	29.5%	16.0%
Bathinda	Punjab	51.4%	7.7%	30.0%	10.9%
Gurdaspur	Punjab	64.9%	0.0%	31.2%	3.9%
Amritsar	Punjab	54.0%	2.6%	35.5%	7.9%
Khadoor Sahib	Punjab	54.4%	3.9%	36.9%	4.7%
Anandpur Sahib	Punjab	54.9%	2.8%	33.7%	8.6%
Sangrur	Punjab	49.0%	5.3%	27.7%	18.0%
Ludhiana	Punjab	51.9%	1.9%	40.4%	5.8%
Faridkot	Punjab	51.4%	8.8%	34.1%	5.7%
Churu	Rajasthan	45.3%	8.2%	41.9%	4.5%
Bikaner	Rajasthan	43.5%	4.5%	50.7%	1.3%
Jhunjhunun	Rajasthan	52.6%	1.8%	40.4%	5.2%
Alwar	Rajasthan	35.8%	5.8%	52.5%	5.8%
Jodhpur	Rajasthan	42.5%	2.9%	50.0%	4.5%
Sikar	Rajasthan	48.9%	6.1%	37.6%	7.5%
Nagaur	Rajasthan	58.8%	4.7%	33.7%	2.8%
Tonk - Sawai Madhopur	Rajasthan	45.4%	4.7%	47.0%	2.8%
Bharatpur	Rajasthan	39.2%	5.8%	49.9%	5.0%
Barmer	Rajasthan	40.5%	2.6%	55.1%	1.8%

Ajmer	Rajasthan	42.9%	5.4%	47.7%	3.9%
Karauli - Dhaulpur	Rajasthan	40.0%	4.5%	52.7%	2.7%
Jhalawar - Baran	Rajasthan	41.9%	1.2%	55.4%	1.5%
Rajsamand	Rajasthan	42.9%	5.4%	49.4%	2.2%
Jalore	Rajasthan	33.1%	2.2%	63.6%	1.0%
Bhilwara	Rajasthan	45.0%	5.4%	48.5%	1.1%
Kota	Rajasthan	38.5%	4.1%	54.5%	2.9%
Pali	Rajasthan	41.0%	4.1%	51.3%	3.6%
Ganganagar	Rajasthan	50.0%	3.3%	45.8%	0.9%
Dausa	Rajasthan	44.1%	6.5%	42.5%	7.0%
Chittaurgarh	Rajasthan	33.3%	1.8%	63.3%	1.5%
Jaipur	Rajasthan	47.4%	9.6%	36.4%	6.7%
Banswara	Rajasthan	25.4%	1.1%	72.3%	1.2%
Udaipur	Rajasthan	29.1%	2.9%	65.9%	2.1%
Jaipur Rural	Rajasthan	46.0%	9.1%	38.3%	6.6%
Sikkim	Sikkim	32.1%	21.6%	18.6%	27.7%
Erode	Tamil Nadu	25.6%	22.8%	23.8%	27.9%
Tenkasi	Tamil Nadu	18.3%	25.3%	18.1%	38.3%
Tirunelveli	Tamil Nadu	18.4%	24.5%	16.3%	40.8%
Kanniyakumari	Tamil Nadu	20.6%	16.2%	13.2%	50.0%
Coimbatore	Tamil Nadu	27.1%	19.8%	22.1%	31.0%
Mayiladuthurai	Tamil Nadu	25.3%	36.4%	15.4%	22.9%
Perambalur	Tamil Nadu	18.4%	24.1%	29.9%	27.5%
Dindigul	Tamil Nadu	20.3%	31.9%	23.2%	24.6%
Arakkonam	Tamil Nadu	17.6%	27.6%	27.1%	27.6%
Chennai South	Tamil Nadu	21.6%	19.2%	28.9%	30.3%
Krishnagiri	Tamil Nadu	21.6%	22.7%	29.6%	26.1%
Arani	Tamil Nadu	15.7%	33.3%	26.7%	24.3%
Tiruvannamalai	Tamil Nadu	14.0%	33.6%	27.3%	25.1%
Sriperumbudur	Tamil Nadu	28.0%	25.8%	18.2%	28.0%
Vellore	Tamil Nadu	15.4%	28.2%	28.2%	28.2%
Kancheepuram	Tamil Nadu	26.8%	26.8%	16.1%	30.4%
Kallakurichi	Tamil Nadu	22.4%	21.7%	27.8%	28.0%
Nilgiris	Tamil Nadu	25.3%	28.0%	21.1%	25.7%
Chidambaram	Tamil Nadu	26.7%	22.5%	29.1%	21.6%
Chennai North	Tamil Nadu	19.6%	16.3%	33.8%	30.3%
Chennai Central	Tamil Nadu	16.6%	13.8%	37.6%	32.0%
Thoothukkudi	Tamil Nadu	31.9%	25.5%	17.0%	25.5%
Nagappattinam	Tamil Nadu	25.0%	28.5%	22.6%	23.8%
Tiruvallur	Tamil Nadu	29.5%	24.8%	20.6%	25.1%
Viluppuram	Tamil Nadu	20.2%	28.3%	26.3%	25.3%
Cuddalore	Tamil Nadu	30.4%	23.2%	30.4%	16.1%
Shivaganga	Tamil Nadu	25.6%	19.8%	15.3%	39.3%
Theni	Tamil Nadu	31.4%	21.7%	20.6%	26.4%
Ramanathapuram	Tamil Nadu	19.0%	20.3%	21.5%	39.2%
Namakkal	Tamil Nadu	20.3%	21.7%	21.3%	36.7%
Thanjavur	Tamil Nadu	20.8%	33.3%	20.9%	25.1%
Tiruchirappalli	Tamil Nadu	19.2%	24.4%	31.7%	24.7%
Pollachi	Tamil Nadu	27.1%	19.8%	22.5%	30.6%
Karur	Tamil Nadu	18.9%	26.9%	26.8%	27.4%

Dharmapuri	Tamil Nadu	23.4%	19.9%	34.4%	22.3%
Madurai	Tamil Nadu	31.2%	20.8%	20.8%	27.1%
Virudunagar	Tamil Nadu	24.2%	24.6%	22.5%	28.7%
Tiruppur	Tamil Nadu	26.6%	23.8%	22.8%	26.8%
Salem	Tamil Nadu	24.1%	16.9%	28.9%	30.1%
Zahirabad	Telangana	43.9%	11.2%	38.5%	6.4%
Khammam	Telangana	44.4%	5.6%	31.5%	18.5%
Medak	Telangana	37.1%	9.7%	45.2%	8.1%
Bhongir	Telangana	37.2%	7.9%	39.9%	15.1%
Chevella	Telangana	46.0%	7.7%	32.3%	14.0%
Secunderabad	Telangana	43.8%	9.8%	25.7%	20.8%
Peddapalle	Telangana	47.4%	10.2%	32.2%	10.2%
Nalgonda	Telangana	32.9%	8.6%	44.3%	14.3%
Nagarkurnool	Telangana	54.2%	3.6%	32.5%	9.6%
Karimnagar	Telangana	52.8%	11.3%	22.6%	13.2%
Nizamabad	Telangana	50.8%	12.2%	29.6%	7.4%
Adilabad	Telangana	38.3%	8.3%	48.3%	5.0%
Mahabubabad	Telangana	44.4%	5.6%	30.5%	19.5%
Mahbubnagar	Telangana	54.2%	3.6%	32.5%	9.6%
Warangal	Telangana	44.4%	5.6%	29.6%	20.4%
Hyderabad	Telangana	43.8%	9.7%	25.8%	20.7%
Malkajgiri	Telangana	45.8%	7.9%	31.9%	14.4%
Tripura East	Tripura	54.4%	4.7%	34.6%	6.4%
Tripura West	Tripura	51.4%	6.5%	27.5%	14.5%
Saharanpur	Uttar Pradesh	52.3%	3.5%	40.4%	3.8%
Kairana	Uttar Pradesh	45.6%	4.3%	45.4%	4.8%
Nagina	Uttar Pradesh	35.5%	2.1%	56.7%	5.7%
Muzaffarnagar	Uttar Pradesh	39.8%	4.7%	49.8%	5.6%
Baghpat	Uttar Pradesh	47.6%	2.5%	46.7%	3.3%
Amroha	Uttar Pradesh	39.9%	3.4%	54.7%	2.0%
Sambhal	Uttar Pradesh	37.4%	5.5%	51.3%	5.8%
Meerut	Uttar Pradesh	44.8%	3.1%	47.2%	4.8%
Lalganj	Uttar Pradesh	45.0%	5.4%	46.5%	3.1%
Jalaun	Uttar Pradesh	28.8%	2.9%	64.7%	3.7%
Rampur	Uttar Pradesh	38.4%	4.9%	53.7%	3.0%
Ghaziabad	Uttar Pradesh	53.2%	1.5%	42.6%	2.7%
Pilibhit	Uttar Pradesh	34.4%	3.0%	60.6%	2.0%
Bulandshahr	Uttar Pradesh	37.7%	4.8%	54.1%	3.4%
Kheri	Uttar Pradesh	28.7%	7.4%	61.0%	2.9%
Bareilly	Uttar Pradesh	37.6%	4.0%	54.0%	4.4%
Aonla	Uttar Pradesh	34.3%	3.5%	58.6%	3.6%
Budaun	Uttar Pradesh	29.1%	2.8%	65.8%	2.3%
Shahjahanpur	Uttar Pradesh	32.6%	7.4%	57.8%	2.2%
Bahraich	Uttar Pradesh	24.8%	6.2%	64.1%	4.9%
Aligarh	Uttar Pradesh	34.6%	3.0%	58.9%	3.6%
Dhaurahra	Uttar Pradesh	30.3%	6.8%	60.2%	2.7%
Etah	Uttar Pradesh	37.3%	3.5%	57.0%	2.2%
Mathura	Uttar Pradesh	46.0%	8.0%	38.7%	7.3%
Farrukhabad	Uttar Pradesh	45.1%	4.0%	47.1%	3.8%
Hardoi	Uttar Pradesh	32.4%	5.9%	55.9%	5.9%

Hathras	Uttar Pradesh	39.2%	4.7%	50.4%	5.8%
Domriaganj	Uttar Pradesh	38.7%	3.9%	54.8%	2.6%
Sitapur	Uttar Pradesh	32.3%	6.2%	59.2%	2.3%
Firozabad	Uttar Pradesh	47.4%	3.3%	44.6%	4.7%
Maharajganj	Uttar Pradesh	31.0%	8.8%	51.4%	8.7%
Mainpuri	Uttar Pradesh	38.3%	4.1%	52.2%	5.3%
Kaisarganj	Uttar Pradesh	28.8%	6.1%	59.3%	5.9%
Gonda	Uttar Pradesh	30.4%	7.2%	56.5%	5.9%
Misrikh	Uttar Pradesh	32.9%	5.9%	56.4%	4.8%
Barabanki	Uttar Pradesh	39.8%	4.9%	51.5%	3.9%
Kushi Nagar	Uttar Pradesh	38.9%	9.4%	41.6%	10.1%
Fatehpur Sikri	Uttar Pradesh	41.9%	4.0%	50.3%	3.7%
Azamgarh	Uttar Pradesh	44.9%	5.4%	46.5%	3.2%
Bansgaon	Uttar Pradesh	42.8%	6.6%	48.4%	2.2%
Amethi	Uttar Pradesh	43.3%	4.2%	49.0%	3.4%
Akbarpur	Uttar Pradesh	36.1%	5.6%	54.0%	4.4%
Rae Bareli	Uttar Pradesh	44.0%	4.3%	50.9%	0.9%
Mohanlalganj	Uttar Pradesh	34.9%	7.5%	57.3%	0.4%
Deoria	Uttar Pradesh	41.9%	7.5%	45.7%	4.8%
Sant Kabir Nagar	Uttar Pradesh	38.3%	5.3%	52.4%	4.0%
Faizabad	Uttar Pradesh	34.3%	2.2%	61.5%	2.0%
Etawah	Uttar Pradesh	35.2%	2.7%	58.2%	3.8%
Sultanpur	Uttar Pradesh	43.0%	4.1%	47.9%	5.0%
Salempur	Uttar Pradesh	41.4%	7.5%	49.1%	2.0%
Ghosi	Uttar Pradesh	42.0%	5.8%	46.5%	5.7%
Chandauli	Uttar Pradesh	38.3%	6.2%	51.0%	4.6%
Allahabad	Uttar Pradesh	26.0%	5.3%	62.6%	6.1%
Mirzapur	Uttar Pradesh	27.6%	9.9%	59.2%	3.3%
Robertsganj	Uttar Pradesh	36.3%	5.4%	55.6%	2.7%
Fatehpur	Uttar Pradesh	35.2%	4.5%	58.0%	2.3%
Jaunpur	Uttar Pradesh	34.6%	7.8%	54.9%	2.6%
Pratapgarh	Uttar Pradesh	45.8%	2.1%	52.1%	0.0%
Hamirpur	Uttar Pradesh	35.9%	1.7%	59.6%	2.9%
Kaushambi	Uttar Pradesh	38.0%	1.4%	60.6%	0.0%
Ballia	Uttar Pradesh	41.3%	6.4%	50.1%	2.2%
Jhansi	Uttar Pradesh	28.6%	8.0%	58.0%	5.4%
Ghazipur	Uttar Pradesh	43.6%	3.8%	51.1%	1.5%
Machhlishahr	Uttar Pradesh	34.4%	7.2%	55.9%	2.5%
Phulpur	Uttar Pradesh	26.0%	5.3%	62.6%	6.1%
Sant Ravi Das Nagar (Bhadohi)	Uttar Pradesh	27.4%	7.0%	59.7%	6.0%
Ambedkar Nagar	Uttar Pradesh	35.7%	5.3%	56.4%	2.6%
Banda	Uttar Pradesh	32.0%	5.7%	58.6%	3.7%
Kanpur	Uttar Pradesh	35.9%	5.7%	54.1%	4.4%
Unnao	Uttar Pradesh	41.8%	2.5%	53.3%	2.5%
Kannauj	Uttar Pradesh	37.3%	7.2%	52.6%	2.9%
Lucknow	Uttar Pradesh	35.3%	7.8%	56.9%	0.0%
Varanasi	Uttar Pradesh	33.5%	5.0%	59.4%	2.1%
Gorakhpur	Uttar Pradesh	41.9%	6.9%	48.1%	3.1%
Basti	Uttar Pradesh	44.6%	3.5%	49.1%	2.8%
Shrawasti	Uttar Pradesh	29.1%	12.1%	54.7%	4.1%

Agra	Uttar Pradesh	42.0%	3.8%	50.8%	3.4%
Gautam Buddha Nagar	Uttar Pradesh	45.5%	4.7%	44.1%	5.6%
Bijnor	Uttar Pradesh	38.4%	3.6%	52.4%	5.7%
Moradabad	Uttar Pradesh	36.8%	4.4%	53.0%	5.8%
Almora	Uttarakhand	38.1%	10.5%	39.9%	11.5%
Hardwar	Uttarakhand	43.0%	8.1%	41.7%	7.1%
Tehri Garhwal	Uttarakhand	32.8%	11.7%	49.1%	6.3%
Garhwal	Uttarakhand	46.3%	8.5%	38.5%	6.7%
Nainital - Udhamsingh Nagar	Uttarakhand	44.7%	9.7%	33.2%	12.3%
Darjiling	West Bengal	45.8%	11.1%	26.0%	17.1%
Arambag	West Bengal	33.8%	19.5%	29.3%	17.5%
Barasat	West Bengal	45.4%	9.6%	17.7%	27.3%
Medinipur	West Bengal	29.1%	20.6%	31.7%	18.6%
Tamluk	West Bengal	31.9%	16.7%	29.2%	22.3%
Murshidabad	West Bengal	30.0%	19.2%	35.8%	14.9%
Krishnanagar	West Bengal	31.4%	20.9%	16.3%	31.4%
Birbhum	West Bengal	29.5%	19.3%	39.8%	11.4%
Bolpur	West Bengal	27.4%	21.4%	35.2%	16.0%
Barddhaman - Durgapur	West Bengal	25.0%	23.7%	30.3%	21.1%
Puruliya	West Bengal	24.6%	24.6%	43.4%	7.4%
Barddhaman Purba	West Bengal	25.0%	23.7%	30.3%	21.1%
Bankura	West Bengal	26.6%	25.5%	33.2%	14.7%
Asansol	West Bengal	25.0%	23.7%	30.3%	21.1%
Ranaghat	West Bengal	31.4%	20.9%	16.3%	31.4%
Bishnupur	West Bengal	26.7%	25.3%	30.9%	17.1%
Jangipur	West Bengal	29.7%	18.9%	39.6%	11.7%
Balurghat	West Bengal	36.5%	12.8%	30.8%	19.9%
Maldah Uttar	West Bengal	34.6%	8.4%	48.6%	8.4%
Kolkata Uttar	West Bengal	41.3%	8.7%	30.4%	19.6%
Jhargram	West Bengal	27.8%	22.1%	34.0%	16.1%
Kolkata Dakshin	West Bengal	36.7%	10.7%	30.8%	21.8%
Uluberiya	West Bengal	33.9%	16.1%	29.0%	21.0%
Mathurapur	West Bengal	26.1%	15.3%	31.5%	27.0%
Jaynagar	West Bengal	34.7%	12.8%	25.4%	27.1%
Diamond Harbour	West Bengal	26.1%	15.3%	31.5%	27.0%
Kanthi	West Bengal	31.9%	16.7%	29.2%	22.2%
Basirhat	West Bengal	47.3%	9.1%	16.4%	27.3%
Bangaon	West Bengal	41.1%	13.7%	16.3%	28.9%
Koch Bihar	West Bengal	30.6%	16.1%	25.8%	27.4%
Alipurduars	West Bengal	24.2%	14.2%	32.7%	29.0%
Jalpaiguri	West Bengal	24.1%	14.2%	32.8%	29.0%
Barakpur	West Bengal	47.3%	9.1%	16.4%	27.3%
Haora	West Bengal	33.9%	16.1%	29.0%	21.0%
Jadavpur	West Bengal	26.1%	15.3%	31.5%	27.0%
Hugli	West Bengal	34.9%	19.0%	28.6%	17.5%
Shrirampur	West Bengal	34.4%	17.6%	28.8%	19.2%
Baharampur	West Bengal	29.7%	18.9%	39.6%	11.7%
Maldah Dakshin	West Bengal	33.5%	10.8%	46.5%	9.2%
Raiganj	West Bengal	35.6%	18.2%	34.6%	11.6%

Ghatal	West Bengal	29.0%	20.8%	31.9%	18.4%
Dum Dum	West Bengal	47.3%	9.1%	16.4%	27.3%
